# Construction and Validation of the Implicit Theories of Sexual Offense Questionnaire (ITSOQ) in a General and (sub)Clinical Population Sample

**DOI:** 10.1177/10790632251326555

**Published:** 2025-04-10

**Authors:** Mirthe G. C. Noteborn, Martin Hildebrand, Jelle J. Sijtsema, Jaap J. A. Denissen, Stefan Bogaerts

**Affiliations:** 1 Tilburg University, Tilburg, The Netherlands; 2 Private Practice, De Bilt, The Netherlands; 3 Lelystad Prison, Lelystad, The Netherlands; 4 University of Groningen, Groningen, The Netherlands; 5 Fivoor Science and Treatment Innovation, Rotterdam, The Netherlands; 6 Utrecht University, Utrecht, The Netherlands

**Keywords:** implicit theories of sexual offending, assessment, questionnaire, antisocial cognitions

## Abstract

This study developed and assessed the psychometric properties of a questionnaire assessing Implicit Theories (ITs) of sexual offense (Polaschek & Ward, 2002; Ward & Keenan, 1999), named the Implicit Theories of Sexual Offense Questionnaire (ITSOQ). We used existing cognition questionnaires to create a potential item pool, and selected items based on item properties (e.g., mean, *SD*, range) from three male general population samples (*n* = 427) and three (sub)clinical population samples (*n* = 69), i.e., pedophilia-supportive forum users (*n* = 20), and sexual (*n* = 28) and violent (*n* = 21) forensic mental health system clients. A principal component analysis for the general population sample supported a four-component solution for the ITSOQ, including two victim-specific ITs (Factor 1: Children 14–16 years, Factor 2: Women), a sexual social desirability index (SSDI; Factor 3), and a component reflecting the antisocial uncontrollability IT (Factor 4). Analyses indicated measurement invariance, and higher scores for the (sub)clinical population were found for the antisocial uncontrollability and SSDI factors, with low to moderate effect sizes. Additionally, (small) associations between self-reported sexual interest in children and adults and the victim-specific child and women factors were found. Implications and directions for future research are discussed.

## Introduction

Cognitions have long been recognized as a critical factor in understanding offense motivation (e.g., Hall & Hirschman, 1991, 1992; Ward & Beech, 2006, 2016). However, disputes persist with regard to the definition, assessment, treatment, and prioritizing of cognitions within the sexual offense chain (e.g., Maruna & Mann, 2006). Ward’s Implicit Theory Model (ITM) of cognitive distortions for men who sexually offended against children (Ward, 2000; Ward & Keenan, 1999) and men who sexually offended against women ( Polaschek & Ward, 2002) is acknowledged as one of the most “rigorous definition of cognitive distortions” in the literature (Mann & Beech, 2003, p. 137). Aligning with classical theories of cognitive psychology (e.g., Kelly, 1955; Levy et al., 1997; Sternberg et al., 1981), Ward and colleagues (Polaschek & Ward, 2002; Ward, 2000; Ward & Keenan, 1999) argued that Implicit Theories (ITs) are networks of interrelated beliefs that shape individuals’ understanding and explaining of their social world. It is hypothesized that the content of these networks influences the interpretation of (interpersonal) situations (e.g., by rejecting or reinterpreting evidence that conflicts with the IT), influence inferences about a third person (e.g., a possible victim), current and future capabilities, goals and desires. Ultimately, these theories may lead to cognitions that facilitate offending (Ward, 2000). Such theories are termed implicit because individuals cannot easily express them as they are rarely formally articulated (Ward, 2000). ITs should be seen as existing on a continuum, and men who (sexually) offended might more strongly endorse one or a combination of ITs. Ward and Keenan (1999) also stated that non-offending individuals may hold these ITs but lack other factors that might lead to offending, such as paraphilic sexual interest and insecure adult attachment.

When differentiating between men who sexually offended against adult women versus men who sexually offended against children, a distinction is made between victim-type (e.g., sexual motivation) versus more general antisocial motivations for offending. Unfortunately, these distinctions have not been accompanied by the development of appropriate assessment methods of ITs. In the present study, we therefore highlight the challenges of using current self-report measures in assessing ITs and, subsequently, developed a questionnaire to address these challenges. This questionnaire includes cognitions that represent IT content and focus on motivations for offending (sexual and antisocial) rather than focusing on the sexual aspect and specific victim types only.

### Implicit Theories in Sex Offending

Examining a variety of cognitions using self-report studies and questionnaire measures to assess the cognitions of men who sexually offended, Ward and colleagues (Polaschek & Ward, 2002; Ward, 2000; Ward & Keenan, 1999) proposed seven core ITs held by men who committed sexual offenses against children and/or adult women. These seven ITs may also be manifest in other offense types as well as in men who never offended. Ward (2000) argued that a man who sexually offended could hold any combination of these ITs. They encompass beliefs about (a) themselves (*Entitlement* and *Uncontrollability*), (b) the nature of their victims (*Children as sexual beings*, *Nature of harm*, *Women as sexual objects*, and *Women are dangerous*), and (c) the world (*Dangerous world*). Among these, the *Entitlement*, *Uncontrollability*, and *Dangerous world* ITs might be more prevalent in men who have sexually offended against children and men who have sexually offended against adult women, and may contribute more specifically to an antisocial orientation. These ITs contain elements of both sexual and antisocial components. Table 1 provides a more extensive description of these seven ITs, including example statements.

**Table 1.** Description of the Seven Implicit Theories.

	Short explanation	Example item	
**The self**			
Entitlement	One feels entitled to act as one pleases due to a perceived superiority over others.	A person should have sex whenever it is needed	Ward & Keenan, 1999, p. 829
Uncontrollability	General beliefs about having no control over life circumstances and behaviors, including sexually abusive actions primarily seen as externally controlled rather than internally controlled. Men who sexually offended against adult women may subscribe to perceptions of uncontrollability linked predominantly to the male sex drive (Males’ sex drive is uncontrollable).	A lot of time sexual assaults are not planned, they just happen	Ward & Keenan, 1999, p. 831
		Men get overpowered by their urges and cannot control their sexual feelings	
**Nature of the victim**			
*Children*			
Children as sexual beings	Children have sexual needs and desires, and actively seek sexual contact.	Children are curious about sex and enjoy it	Ward & Keenan, 1999, p. 828
Nature of Harm	Sexual activity alone is unlikely to harm children, with harm arising only when accompanied by force or threat.	Many children who are sexually assaulted do not experience any major problems	Ward & Keenan, 1999, p. 832
* Adult women*			
Women as sexual objects	Women constantly desire sex, even if forced or violent.	Rape of a woman by a man she knows can be defined as ‘a woman who changed her mind afterwards	Polaschek & Ward, 2002, p. 396
Women are dangerous^a^	Inherent differences exist between men and women, with men struggling to understand these differences.	Most women are sly and manipulating when they are out to attract a man	Polaschek & Ward, 2002, p. 396
**The world**			
Dangerous world	The world is a dangerous place in which others are malevolent and aggressive. Some men offending against children exempt children from this belief, seeing children as dependable, caring and trustworthy.	Lots of people are out to get you Children are innocent and want to please adults	Polaschek & Ward, 2002, p. 399 Ward & Keenan, 1999, p. 830

^a^This IT was previously termed Women are unknowable (Polaschek & Ward, 2002).

### Self-Report Questionnaires to Assess Cognitions in Sexual Offending

Accurately assessing the cognitive distortions and ITs of men who sexually offended has proven to be challenging. Typically, assessments employ self-report questionnaires or clinical interviews (e.g., Polaschek & Ward, 2002). Over the years, several self-reports have been developed to assess the cognitions of men who sexually offended against children or adult women (e.g., RAPE and MOLEST scale; Bumby, 1996). Ward and colleagues (Polaschek & Ward, 2002; Ward & Keenan, 1999) used corresponding items from these cognition questionnaires to identify the seven ITs described earlier. However, using these questionnaires to measure ITs has serious shortcomings when applied to populations of men who sexually offended. For example, cognition questionnaires contain statements that do not necessarily reflect the identified ITs (e.g., “I never sulk when a woman makes me angry” from the Hostility Towards Women (HTW) scale [Check et al., 1985; Gannon et al., 2008; Hermann et al., 2012). We acknowledge terminological discussions and complexities in discussing cognitions in sexual offending (e.g., Ó Ciardha & Gannon, 2011). Regarding ITs, we refer to cognitions reflecting long-term cognitions that precede or facilitate offending by guiding an individual down a long-term path (Szumski et al., 2018). However, current questionnaires often include short-term pre-offense cognitions that provide proximal justification of offense activity and post-offense rationalizations (for an overview of distorted cognitions related to male sexual offending, see Szumski et al., 2018; for an overview of post-offense justifications and excuse-making, see Maruna & Mann, 2006).

Another shortcoming of existing cognition questionnaires is that they overlook specific antisocial ITs, which is unfortunate given their important role as a risk factor for both sexual and general reoffending (e.g., Hanson & Morton-Bourgon, 2004, 2005). Although assessing ITs that center on a sexual theme seems logical for men who sexually offended, the vast majority of men who sexually offended are generalists (i.e., they commit a wider range of crimes than just sexual offenses), suggesting the presence of general antisocial features (e.g., Harris et al., 2009). In addition, research has suggested that many men who committed sexual offenses do so against both children and adults (also known as age cross-over offending; Heil et al., 2003; for a comprehensive review, see Saramago et al., 2020). However, many classifications of men who sexually offended are based solely on the nature and characteristics of the specific offense for which the person is currently incarcerated or arrested (i.e., index offense). These classifications overlook cross-over offenses and victim characteristics over extended periods (Cale, 2018). Since sexual offending against both adults and children may indicate a more general antisocial and pro-criminal motivation instead of specific dysfunctional sexual scripts (e.g., Ward & Siegert, 2002), it could be argued that some of the men who sexually offended and harbor a more general antisocial motivation for sexual offending may leave treatment with important criminogenic factors unaddressed due to possibly inadequate assessment (Beech et al., 2013).

### Item Endorsement

An important challenge in using cognition questionnaires in order to measure ITs, is the low base rate of these cognitions (e.g., Merdian et al., 2014). When assessing discriminative validity among specific sexual offense types (i.e., against children/women) and control groups, it would be accurate to state that the target population of men who sexually offended tends to *disagree less* with these cognitions rather than displaying a difference in the presence of these cognitions (e.g., Tierney & McCabe, 2001). The low base rate of affirmative responses on these self-reports often confines respondents to endorsing only the lowest options of ‘strongly disagree’ and ‘disagree’ on a Likert scale. This makes the clinical relevance of such a one-point difference questionable (e.g., Steel et al., 2020).

In line with the low base rates of endorsement, particularly in socially sensitive domains (e.g., sex with children, being entitled to sexual contact regardless of a woman’s feelings; Gannon et al., 2008), questionnaires used to measure IT content are susceptible to social desirability biases and manipulation (e.g., Gannon, 2006). A potential solution to this problem involves the administration of an existing self-report that measures the (general) tendency to present oneself more favorably (i.e., impression management) concurrently with IT questionnaires. Although a separate questionnaire to assess social desirability may be helpful, the tendency to be open about general flaws (gossiping, covering up mistakes) does not necessarily predict, for example, honesty in responding to questions about children and sex (Keown et al., 2010). Including a ‘social desirability index’ in the IT questionnaire, consisting of questions related to the self-report theme, could be beneficial because it more directly addresses the answering tendency of the target questionnaire.

### Current Study

Given the challenges in current questionnaires assessing ITs, we aimed to develop a questionnaire to measure the seven ITs related to sexual offending as proposed by Ward and colleagues (Polaschek & Ward, 2002; Ward, 2000; Ward & Keenan, 1999). This new self-report questionnaire should address the previously mentioned limitations, incorporating only cognitions that represent IT content. It focuses on motivations for both sexual and antisocial offending rather than solely focusing on the sexual aspect of the crime and specific victim types. Additionally, a sexual social desirability index was developed and integrated into the new questionnaire.

We chose an explorative factor approach for the development of the questionnaire, as it provides a basis for removing redundant or unnecessary items (Anthony, 1999), and also can identify the underlying domains within a selected item pool (Ferguson & Cox, 1993; Oppenheim, 2000). Following the recommendation to use existing validated instruments in questionnaire development (e.g., Fayers & Machin, 2007), and acknowledging that existing cognition questionnaires contain IT content (e.g., Gannon et al., 2008), the development of our new questionnaire, which we named the Implicit Theories of Sexual Offense Questionnaire (ITSOQ), drew partly from items from existing self-report measures assessing the cognitions of men who sexually offended against children or against adult women, supplemented by example items provided by the original authors (Ward and colleagues) of the seven ITs. Since there are considerable similarities between the seven ITs (e.g., Steel et al., 2020), we did not expect that the EFA would result in a seven factor solution with the seven factors representing the seven ITs. For example, there is interdependence between certain components, such as thinking that children are capable of making informed decisions about sexuality, as conceptualized in the *Children as sexual beings* IT, and the idea that some children seek sexual contact with adults for affection, as conceptualized in aspects of the *Dangerous world* IT. In addition, researchers have already stated that, for instance, parts of the IT *Nature of harm* work in unison with the *Children as sexual beings* IT (e.g., Gannon & Polaschek, 2006).

The exploratory approach was followed by multi-group confirmatory factor analysis (MG-CFA) to investigate measurement invariance. This ensures that observed variations reflect actual group differences, confirming consistent construct interpretation across groups (Putnick & Bornstein, 2016). Without obtaining measurement invariance, group comparisons become uncertain, as disparities may stem from psychometric variations in item responses rather than differences in the fundamental construct.

Finally, after creating our questionnaire, group differences between different (sub)clinical populations and the general population were investigated. Because we did not expect to find all seven IT as individual components, a detailed a priori hypothesis could not be formed. The general hypothesis was that higher scores on offense related factors will be found for the (sub)clinical population compared to the general population. Additionally, we aimed to validate IT scores by correlating scores with a self-report measure that captures the degree of sexual interest towards children and adults. Sexual interest in children is one of the best predictors of sexual recidivism among men who sexually offended against children (Hanson & Morton-Bourgon, 2005). As the literature suggests overlap between sexual interest in children and the *Child as sexual beings* IT (Ó Ciardha, 2011), and the involvement of ITs in paraphilic sexual interest (Ward & Beech, 2006), we hypothesized that a positive association between higher scores on factors related to sexual contact with children and an increased degree of sexual interest in children will be found.

## Method

### Participants and Procedure

Data collection was carried out in several samples. Ethical approval was obtained for all samples. In Table 2, procedural and demographic information of all samples is provided.

**Table 2.** Descriptives of Demographic Information and Data Collection Procedure of the General and (sub)Clinical Population Samples.

Population		Specific population				
Description	*N*	Description	n	Age (M, SD) (missings)	Ethical Approval	Received compensation
General population	427	Online survey	148	33.2 (15.56)	Ethical Review Board Tilburg University TSB_RP590	No^1^
		Paper and pencil	219	33.9 (13.99) (14)	Medical Ethical Committee (METC) NL55030.028.15/P1546	Voucher €10,-
		Lab	60	30.2 (13.57)	Medical Ethical Committee (METC) NL55030.028.15/P1546	Voucher €10,-
(Sub)Clinical population	69	Offended violently	21	37.0 (10.49)	Medical Ethical Committee (METC) NL55030.028.15/P1546	Voucher €10,-
		Offended sexually	28	46.7 (12.03) (1)	Institutional Review Board VIGO Group (HREC, 2021–001)/Medical Ethical Committee (METC) NL55030.028.15/P1546	No/ Voucher €10,-^2^
		Pedophilia-supportive forum users	20	37.7 (11.62)	Ethical Review Board Tilburg University TSB_RP590	No^1^

*Note*. All data collection included several additional measures, besides the ITSOQ and the BIDR.1For the online questionnaire via Qualtrics data collection, compensation of participants was not possible due to anonimity. 2Depending on clinic (ethical approval).

#### General Population

In total, data from three male population samples (*n* = 427) were collected by bachelor and master level students by convenience sampling. Participants were asked to fill out questionnaires (1) at home and return them within sealed envelopes; (2) in a lab setting with the first author present; or (3) via an anonymous online link. Participants were informed that the current research aimed to gain insight into thoughts about sexuality and intimacy, and how these thoughts relate to behaviors and individual characteristics in various (target) groups. Besides standard requirements (e.g., the possibility of withdrawal, time to reflect on participation), several safeguards were put in place to further minimize potential emotional harm of the study. Participants were discouraged to participate if they were themselves victims of sexual abuse, informed about possible emotional discomfort due to the topic of the study, and given the opportunity to consult an independent psychologist. To inform and, if needed, support participants, information was given about sexually aggressive behavior and treatment options for both victims of sexual abuse and men who sexually offended, including contact information for further support after completing the study. None of the participants made use of these additional safeguard measures, and no negative consequences were reported or observed.

On average, participants from the general population were 33.1 years old (*SD* = 14.5; range 18–87). Results of Kruskall-Wallis analysis (necessary because of non-normal distributions), showed a significant age difference between the three subsamples, *H*(2) = 11.108, *p* < .001, ε^2^ = .03, using Holm adjustment to correct for multiple testing. Participants in the lab-setting were significantly younger than the participants in the paper-and-pencil group, *U* = 4500.5, *p* = .005, *r* = −.19, AUC = .34.^1^

#### (sub)Clinical Population

Data from three (sub)clinical population samples were collected. For the purpose of the study, we labeled these populations as (sub)clinical populations, as we hypothesized that these populations would have higher scores on (specific) ITs than the general population.

##### Pedophilia-Supportive Forum Users

Male users from the online Dutch forum for pedophilia (*n* = 20) (https://www.pedofilie.nl/) were recruited with the consent of the moderators of the forum.^2^ The forum is intended for and created by people with pedophilic feelings, providing information and a platform for discussing pedophilic feelings with others. The information letter and link to the online questionnaire were provided by the first author and moderators of the forum in the weekly (publicly accessible) interactive chat of the forum. Participants were asked to complete an anonymous online questionnaire. For debriefing, participants were given information about sexually aggressive behavior and treatment for victims of sexual abuse and men who have sexually offended, including contact information for further support after completing the study (i.e., contact information of the first author and a link to https://www.stopitnow.nl/).

Participants from the online forum had an average age of 37.7 (*SD* = 11.62; range 22–57). Of these pedophilia-supportive forum users, eight users had undergone treatment for pedophilic feelings, with two being mandated by court. Additionally, six forum users reported having been in contact with law enforcement, with five of them related to their pedophilic feelings (e.g., downloading and distributing child pornography, child sexual abuse).

##### Men who Sexually or Violently Offended

Forty-nine men residing in two forensic psychiatric treatment institutions in the Netherlands were recruited for participation and asked to complete a questionnaire. These men were residing in either a forensic psychiatric center or a forensic psychiatric department (i.e., among others, there is a difference in level of security). All participants received inpatient treatment mandated by a court order (terbeschikkingstelling [TBS]) because a causal connection has been established between the person’s mental health status and their offense. TBS is a provision in the Dutch criminal code that allows protection of society in the short term by detention, followed by a period of treatment to reduce risk in the long term (Van Marle, 2002). Twenty-eight of the men residing in forensic inpatient facilities were in mandatory treatment for sexual offenses; 15 for contact sexual offenses with minors, nine for contact sexual offenses with adults (e.g., rape), and four for child pornography.

The average age of the men who sexually offended was 46.7 years old (*SD* = 12.03; range 26–65). Additionally, 21 men in mandatory treatment for violent crimes (e.g., manslaughter, aggravated assault, murder) participated in the study, with a mean age of 37.0 years old (*SD* = 10.49; range = 24–58). Mann-Whitney U tests revealed that the (sub)clinical population (*M* = 41.1, *SD* = 12.20) was significantly older than the general population sample (*M* = 33.1, *SD* = 14.53, *U* = 7850, *p* < .001, *r* = −.26, AUC = .27). Results of Kruskal-Wallis analysis indicated significant age differences between all samples, *H*(3) = 37.85, *p* < .001, ε^2^ = .08. The general population sample (*U* = 2308.50, *p* < .001, *r* = −.23, AUC = .19) was significantly younger than the sample of men who sexually offended. In all analyses, we applied Holm’s correction (Holm, 1979) for multiple comparisons.

### Measures

#### Development of the Implicit Theory of Sexual Offense Questionnaire

**Figure 1.**
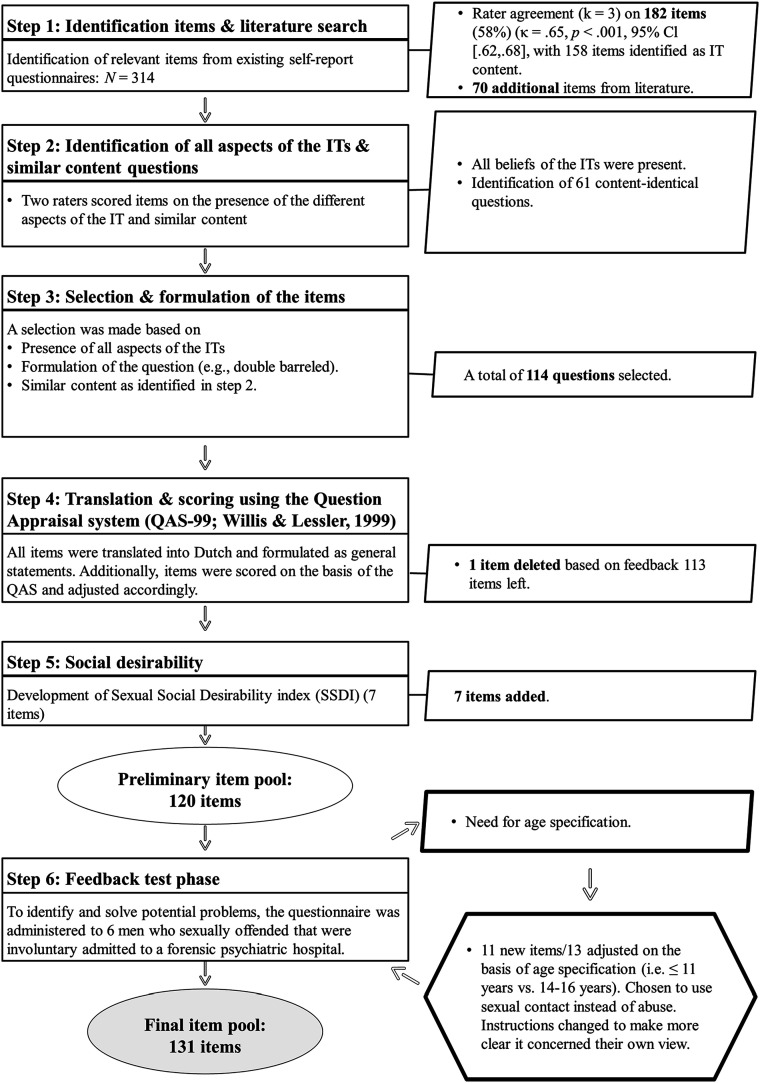
Steps in the Development of the Implicit Theories of Sexual Offense Questionnaire.

The development of the ITSOQ consisted of several steps, outlined in Figure 1. For a detailed description of the development of the ITSOQ we refer to the online supplemental material.

First, to identify items that fit Ward and colleagues’ seven core ITs (Polaschek & Ward, 2002; Ward, 2000; Ward & Keenan, 1999), three of the authors independently mapped all items from existing cognition questionnaires^3^ (in total 314 items) into the respective ITs (cf. Gannon et al., 2008). Only items on which the three raters had complete agreement were selected, resulting in a total of 182 items, of which 158 were classified as belonging to an IT. As expected, and in line with Gannon et al. (2008), results from the first step indicated that items reflecting the three general antisocial ITs were underrepresented. Only for the *Entitlement* IT, general antisocial items were identified. Therefore, we also searched the literature for example items of the ITs as expressed by Ward and colleagues. This resulted in an additional 70 items.

In step two, it was checked if all aspects of the specific ITs were covered in the selected items. For example, the *Dangerous world* IT consists of (1) the belief that the world is hostile and people behave in an abusive and rejecting manner, and (2) of the belief that adults are unreliable, and children are dependable. We also checked whether items had the same content (for example, “Some children act seductively towards adults” and “Some children can act very seductively”). Sixty-one items reflecting the same content were identified by two of the authors in agreement.

Furthermore, because the ITSOQ is intended to assess ITs of men who sexually offended against children as well as the ITs of men who offended against adult women, victim-unspecific items (23 items) were modified to align with victim-specific ITs (e.g., *Children as sexual beings*, *Women as sexual objects*). Therefore, to increase the number of offense-specific items, the victim-unspecific items were transformed to characterize one of the two offense categories (e.g., “She is flirting and teasing me, so she wants to do it” became “If a child is flirting and teasing, it wants to have sex”).

In step three, an item selection was made based on the results of Step 2 (i.e., all aspects IT content present, limiting the number of content-identical questions), and the formulation of the question (e.g., double-barreled items were deleted due to possible bias), resulting in an item pool of 114 items.

In step four, the selected items were back-translated into Dutch and formulated as general statements, as this is considered the best way to tap into IT constructs (e.g., Gannon & Polaschek, 2006). To eliminate biases in item formulation (for an overview of possible biases, see Choi & Pak, 2005), all items were reviewed by three of the authors using a questionnaire appraisal system (QAS; Willis & Lessler, 1999). The QAS can identify potential problems in the wording or structure of the items by checking features that are likely to cause problems (e.g., technical terms, vague items, undefined and unclear standard terms, double-barreled items). Items were adjusted in such cases. Also, a five-point Likert-type scale was developed, with scores ranging from 1 (*disagree)* to 5 (*agree*) with a neutral midpoint. Although using a neutral midpoint is not always optimal as it does not indicate a preference (e.g., Krosnick et al., 2002), we believe that in the case of sensitive topics, a neutral answer might be considered a more ‘safe’ option for participants harboring the specific cognition even though it is also a response that can indicate a deviation from the norm (e.g., being neutral to sexual contact with a minor). Feedback on the selected items was provided by several professionals from different fields. One item was removed due to its resemblance to another item, resulting in an item pool of 113 items.

Next (step five), we developed a social desirability scale that focused on sexual content, which we named the Sexual Social Desirability Index (SSDI; e.g., “Most men masturbate”). The SSDI consists of seven items with sexual content that would be expected to be answered conservatively, based on social norms. That is, one normally does not openly discuss such relatively sensitive topics. Higher scores on the SSDI indicate less socially desirable answering.

To identify and solve potential problems, Step 6 tested the preliminary 120-item version of the ITSOQ in a pilot. Seven items were added twice in the item pool to assess the impact of formulation and specificity on responses patterns. Four items were included twice using terms like abuse/offense, versus using the term sexual contact (e.g., “If a person does not use force when *sexually abusing* a child, it will not harm the child as much” vs. “If a person does not use force to *have sexual contact* with a child, it will not harm the child as much”). Furthermore, three items introduced an age specification, distinguishing children aged 13 years and younger as a category versus no age specification category. An example of the former is “A child aged 13 years or younger can make their own decisions as to whether she (he) wants to have sex with an adult or not”. An example of the latter is “A child can make their own decisions as to whether to have sexual activities with an adult or not”.

The preliminary item pool was administered to six males who sexually offended (based on index offense) and were involuntary admitted to a forensic psychiatric hospital (five of them with age cross-over offending). The main feedback concerned the lack of an age specification in the *Children as sexual beings* and *Nature of harm* ITs. All participants indicated responding conservatively on these ITs, recognizing that, for instance, a seven year old child does not have the cognitive capacity to decide whether or not to have sexual contact, but this might be different for a 12-year old child. However, participants did not seem to differ in their scoring when looking at the items with age differentiation. Participants indicated an cut-off of 14 years as appropriate. For this reason, the items in which the age of the child could influence the response of the participant (e.g., “If a child flirts with men, this means that the child wants to have sexual contact”) were formulated in relation to children between 14-16 years versus children aged 11 years and younger. These age categories aimed to eliminate possible ambiguity in sexual maturation (i.e., 12–13 years). However, after looking at the questions, it appeared that only the age category of children aged 11 years and younger was suitable for some items, as using an older age category would be questionable. For instance, “A child that walks around naked is instigating sexual contact” seems to imply a lack of awareness of the social consequences of being naked, which generally speaking does probably not apply to children older than 14. Additionally, as the items were formulated as general statements, some participants tended to answer questions based on general societal view, particularly with regard to having sexual contact with children. Therefore, we explicitly instructed participants to respond according to their individual perspectives. The final item pool comprised 131 items.

#### Sexual Interest

To assess sexual interest, participants rated their level of interest in males and females across age categories (i.e., 18 years and older; 14–16 years; 12–13 years; 11 years and younger) on a scale from 0 (*no interest at all*) to 100 (*strong interest*). These categories corresponded with the age distinctions in item content as well as Dutch legal definitions. The age category of 17 years was omitted for clarity in differentiation between categories. Except for one sample of men who sexually offended (Data collection only included the social desirability measure and the initial item pool of the ITSOQ), all samples completed this questionnaire.

#### Social Desirability

To control for socially desirable responses and to assess the validity of the SSDI of the ITSOQ, a short form of the Balanced Inventory of Desirable Responding (BIDR; Paulhus, 1984) was used, the 20-item dichotomously scored BIDR-D20 (Noteborn et al., 2024). In the BIDR-D20, social desirability consists of two constructs: impression management (IM) and self-deception enhancement (SDE) (Li & Bagger, 2007; Paulhus, 1988). IM refers to the tendency to intentionally distort one’s self-image to be perceived favorably by others (e.g., “When I hear people talking privately, I avoid listening”). SDE, on the other hand, is an unintentional tendency to portray oneself in positively biased but honestly believed self-descriptions (e.g., “I always know why I like things”). The items are presented as statements, and participants indicated agreement on a 7-point Likert scale (1 = *totally disagree*, 4 = *neutral*, 7 = *totally agree*). Internal consistencies of the BIDR-D20 IM (*ω*general population sample = .76; *ω*(sub)clinical sample *=* .68) and SDE (*ω*general population sample = .70; *ω*(sub)clinical sample *=* .83) were comparable with results of previous studies on the original BIDR (e.g., Li & Bagger, 2007). The internal consistency of the IM and SDE subscales for the general population samples in the current study was slightly lower than the levels reported in the original study on the BIDR-D20 (ω > .78; Noteborn et al., 2024).

### Analytic Approach

To reduce complexity and improve scale properties, we first eliminated items with undesirable descriptive properties across samples. Specifically, we based decisions on whether or not to remove items on the item means, standard deviations, and the range. Given low base-rate of endorsement of these cognitions (e.g., Merdian et al., 2014), selecting items with desirable distributions becomes important. Therefore, items were favored if they had means between 2–4, a *SD* > .5, and covered the entire range.

Second, Principal Component Analyses (PCA; Netemeyer et al., 2003) was conducted to remove items with unclear loadings. The PCA was performed using data from the general population sample. Although simulation studies demonstrate that PCA can be feasible (e.g., simple factor structures) with sample sizes as low as *N* = 50 in certain circumstances (e.g., de Winter et al., 2009), we decided against this approach due to the high ratio of initial items, the possible complex factor structure, and the small sample size of the (sub)clinical population sample.

Because it was assumed that factors would be correlated, an oblimin rotation (Jennrich & Sampson, 1966) was employed. Factor intercorrelations with obliquely rotated results were examined to determine if oblique rotation was justified. Both the pattern and structural matrix were examined (Henson & Roberts, 2006). The number of components was determined using the scree test (Cattell, 1966) in which interpretation requires some level of subjective judgement, combined with parallel analysis (Horn, 1965; Turner, 1998). Parsimony, theoretical convergence and reliability of the scales were also considered as criteria.

After determining the number of components, items were retained that showed a robust loading on one component while exhibiting small or zero loadings on others. Recommendations for cut-offs for primary component loadings to be considered acceptable vary, ranging from .32 for samples over 300 (Tabachnick & Fidell, 2007), > .40 (Stevens, 2002), to values of .60 or.70 (Matsunaga, 2010). As the goal of the PCA was to select the best items while retaining sufficient items per scale, we prioritized primary loadings > .40. Subsequently, secondary component loadings were examined (i.e., the item’s second highest loading, also called cross-loadings), flagging items as problematic if the secondary loadings were > .30 (Howard, 2016). Lastly, attention was given to the discrepancy between the primary and secondary component loadings, with items flagged as problematic if the primary-secondary discrepancy was too small (<.20; Howard, 2016).

After item selection based on PCA, we tested measurement invariance across the general and (sub)clinical populations using structural equation modeling (SEM) and MG-CFA. In MG-CFA, the rule of thumb is 100 cases/observations per group (Kline, 2010). However, parameter estimates are thought to be essentially unbiased if models converge and have a proper solution (Chen et al., 2001). Measurement invariance testing involved a stepwise hierarchical comparison of increasingly constrained models (Vandenberg & Lance, 2000). As a first step, a baseline model is fit to the data in which the loading pattern is similar across groups, allowing other parameters (e.g., loadings, intercepts, and variances) to vary. Second, configural invariance is tested. Configural invariance indicates that the baseline model has a good fit and the same loadings are significant in all groups (Putnick & Bornstein, 2016). After establishing configural invariance, metric invariance (also called weak invariance) is tested. Metric invariance indicates that the magnitude of the loadings is similar for the general and (sub)clinical population. The final step in testing measurement invariance is scalar invariance (or strong invariance), where factor loadings and item intercepts are constrained to be equivalent across the general and (sub)clinical population samples. Scalar invariance indicates no systematic response biases, allowing for mean comparison between the general and the (sub)clinical population sample (Putnick & Bornstein, 2016). The final step in establishing measurement invariance is testing for invariance in the residuals, also called residual or strict invariance. Obtaining residual invariance indicates that the variance of the item that is not shared with the factor and error variance (measurement error) is similar across the general and (sub)clinical population sample. Though strict invariance is considered the final step in testing measurement invariance, it is considered inconsequential to interpretation of the latent mean differences (e.g., Vandenberg & Lance, 2000).

Configural invariance is tested by evaluating the overall fit of the model. For all other forms of measurement invariance, if the overall model fit is not significantly worse than the model tested in the previous step, it indicates that constraining the specific parameter (i.e., loadings, intercept, variance) did not significantly affect model fit, thus supporting that form of variance (Putnick & Bornstein, 2016)

The maximum likelihood with robust standard errors (MLR) estimator was used to account for non-normal distributions (Muthén & Muthén, 1998- 2017–2017). The overall model fit of the baseline and configural model was assessed as follows: the root mean square of error of approximation (RMSEA) with values of ≤ 0.10 indicating an acceptable fit, values of ≤ 0.08 suggesting an approximate model fit, and values of ≤ 0.05 pointing to a good model fit (Chen et al., 2008). The standardized root mean square residual (SRMR) with values of ≤ 0.08 were used for determining a good model fit (Hu & Bentler, 1999) Additionally, the comparative fit index (CFI) with values of ≥ 0.90 were used (Hu & Bentler, 1999).

Nested model comparisons involve computing the difference between fit statistics for the different invariance models. We followed the guidelines of ΔCFI < .010, ΔRMSEA < .015, and ΔSRMR < .030 (e.g., Little, 2013) for accepting invariance. Due to unequal groups sizes and the fact that the size for the (sub)clinical sample was rather small (*n* = 69), we also looked at the decision rules ΔCFI ≤ .005 and ΔRMSEA ≤ .010 (Chen, 2007). Because the Satorra-Bentler χ^2^ (S-B χ^2^; Satorra & Bentler, 2001) statistic is sensitive to sample size and a statistically significant Δχ2 can even occur when there are only minor differences between the general and (sub)clinical factor patterns, we only used the S-B χ^2^ and the S-B χ^2^ difference test (ΔS-B χ^2^) for descriptive purposes (e.g., Chen, 2007; Cheung & Rensvold, 2002). The authors take responsibility for the integrity of the data, the accuracy of the data analyses, and have made every effort to avoid inflating statistically significant results. Main analyses were prespecified prior to data collection. We report on the sample size(s), all data exclusions and all measures in the study.

All analyses were conducted using R version 4.2.2 (R Core Development Team, 2022). Descriptives, correlational analyses, and PCA were conducted using the package Psych (Version, 2.3.3; Revelle, 2018). Parallel analyses and MG-CFA were performed using the packages Paran (Version 1.5.2; Dinno, 2018), Lavaan (Version 6.15; Rosseel, 2012) and SemTools (Version 5.6; Jorgensen et al., 2022), respectively.

## Results

### Item Selection: Item Means, Standard Deviations, and Range

Item means, *SD*s and ranges were examined using the criteria outlined in the method section. Each criterion that was not met in one of the samples (normal population sample, pedophilia-supportive forum users, sexual offending sample, violent offending sample), was marked as “problematic”. In total, 19 items met all the criteria in every sample and were retained for further analysis. To make a further selection of the items, items were retained in the item pool if means in all samples were acceptable (*M* ≥ 1.50), with at least one *M* > 2.00 in one of the samples, and *SD* (>.50) and range (≥3).This resulted in an additional 31 items. Also, four items were retained in the analyses as means were considered acceptable to appropriate across samples, with satisfactory *SD* (>.50), but with a range of 2 in the violent offense sample. This resulted in a total number of 54 IT items. Means, standard deviations and ranges of the complete item pool can be found in the online supplemental material.

Regarding the items of the SSDI, all seven items had high mean scores (>3.52), with most scores across samples of *M* > 4.00. As the goal of the SSDI items was to mark only the respondents that have a tendency to answer in a socially desirable manner, high scores were expected. With the exception of the pedophilia-supportive forum users (*SD* > .40), *SDs* were adequate (>.50). Ranges were adequate in the general population sample, but lower in the other samples. For now, five SSDI items were retained for further analysis as criteria of these items were acceptable across at least two samples.

Taking a closer look at the items selected, for the *Entitlement* and *Women as sex objects* ITs, only two and three items were present, respectively. When looking at the difference between the items with the different age categories (≤11 years vs. 14–16 years), Wilcoxon signed-rank test due to non-normal distributions analyses showed that the general population sample and the men who violently offended indicated less disagreement with items referring to 14–16 years (i.e., higher scores but still on the disagreeing end). The forum users and the populations of men who sexually offended showed less disagreement with the items when the age category 14–16 years was used or indicated no differences in (dis)agreement (see online supplemental material for the analyses).

### Principal Component Analysis

The 59-item set (54 IT items and 5 SSDI items) was analyzed using PCA followed by oblique rotation within the general population sample. A detailed overview of all the analyses can be found in the online supplemental material. PCA was performed using a correlation matrix with Spearman correlations as Mardia’s multivariate skewness and kurtosis indicated multivariate non-normality (*p* < .001; Watkins, 2018). In total, 0.5% of the data was missing, and this was handled using mean imputation in the PCA analysis (Schumacker, 2015). To assess item redundancy and relatedness, inter-item correlations were assessed. When inspecting the correlation matrix, one item had high inter-item correlations (*r* > .70) with two other items, indicating redundancy (Polit & Beck, 2008). For this reason, this item was removed from the analyses. Bartlett’s (1954) test of sphericity indicated that the correlation matrix was not random, χ2(1653) = 7562.436, *p* < .001, and the Kaiser-Meyer-Olkin (KMO) measure of sampling adequacy (Kaiser, 1974) was .86 (KMO item level .58–.92), which is well above the minimum standard for conducting PCA (Tabachnick & Fidell, 2007). It was therefore determined that the correlation matrix was appropriate for PCA (Netemeyer et al., 2003).

With regard to component-retention, although parallel analysis suggested that six components were required, four components could be observed from the scree plot (first 10 eigenvalues: 9.67, 3.85, 3.63, 1.95, 1.79, 1.61, 1.54, 1.39, 1.33, 1.27). Therefore, the four-, five- and six-component solutions were sequentially examined. The scree plot and the results of the parallel analyses can be found in the online supplemental material.

First, all components intercorrelated, mean *r* four-component = .13 (range .00–.35); mean *r* five-component = .13 (range .02–.34); mean *r* six-component = .12 (range .02–.33), therefore oblique rotation was supported. When comparing the pattern and the structure matrix, results showed that for all three component solutions, the items were stable regarding their component membership. Secondary loadings, however, were overall higher in the structure matrix (both pattern and structure matrices of all component-solutions can be found in the online supplemental material).

The five- and six-component solutions were considered inadequate as they contained too few items to be considered valid and did not form internally consistent scales. Therefore, the five- and six component models resulted ipso facto in a four-component solution with a structure similar to the original four component model. The four-component model showed a clear solution based on component loadings criteria described a priori (primary > .40, secondary < .30, Δ primary - secondary < .20) and was regarded as the optimal solution. The first component consisted of 13 items with component loadings between .42–.82. After rotation, the first component accounted for 11% of the variance. The items of this component represent a mixture of items that would fit the *Children as sexual beings* and *Nature of harm* ITs, except for one item reflecting a sexual uncontrollability content. Ten items referred to children in the age category of 14–16 years, two items did not make a reference to age and one item referred to 11 years and younger. As we aimed to create scales with a maximum of 10 items and if the four-component model would be the most adequate structural representation of the data, three items should be deleted. Looking at item content and component loadings (i.e., with the lowest loadings), the items referring to the age category 14–16 were retained in the scale, resulting in a total of 10 items with an internal consistency (alpha) reliability of .89 (95% CI = .87–.89).

The second component consisted of 12 items and accounted for 10% of the total variance. Ten items were related to untrustworthiness and complexity in the understanding of women and two items were representative of a more general selfishness of people. Taking into account parsimony, theoretical convergence, and factor loadings (i.e., items with the lowest loadings), the items reflecting untrustworthiness and complexity in the understanding of women were retained, resulting in a 10 item component with an internal consistency of .83 (95% CI = .80–.85).

The third component contained five items; four SSDI items and one item with a negative component loading related to sexual contact with a child (<11 years). The third component accounted for 6% of the total variance. In order to keep the third component thematically consistent, the item regarding sexual contact with a child was removed. Thus, the third component consisted of four items with an alpha coefficient of .69 (95% CI = .64–.74).

The fourth component contained four items, all reflecting a sense of uncontrollability in life. The component accounts for 5% of the total variance with an alpha coefficient of .66 (95% CI = .60–.71).

In sum, the final four-component solution consisted of 28 items and contained three IT scales, two reflecting victim-specific content, namely children (component 1) and women (component 2), and one representing general uncontrollability (component 4). The other component reflected a social sexual desirability index. All components had high component loadings (.48–.81) and acceptable to optimal internal consistencies (DeVellis, 2003; Nunnally & Bernstein, 1994) for research purposes. However, according to certain standards, internal consistency would be too low for clinical practice (Bland & Altman, 1997). Table 3 presents the final four-component solution (with sample items translated into English).

**Table 3.** Results of the Final Components of the Principal Component Analyses in the General Population Sample (*N* = 427).

Nr	Item Description	*M*	*SD*	Component Loadings				h2	Δ Primary- Secondary Loading
				C1.	C2.	C3.	C4.		
1	Children (14–16 years) who have been involved in sexual activities with an adult male, will eventually get over it and get on with their lives	2.07	1.06	**.63**	.09	.04	−.12	.46	.51
2	It is OK to have sexual contact with a child (14–16 years) as long as the child is not forced into it	1.61	0.97	**.70**	−.05	−.10	.04	.47	.60
3	Some children (14–16 years) are mature enough to enjoy sexual activities with men	2.18	1.20	**.81**	−.04	.04	.03	.64	.77
4	A child (14–16 years) can make its own decisions as to whether or not to have sexual contact with a man	1.95	1.02	**.69**	.08	−.01	.12	.55	.57
-	Some men are not “true” child molesters - they have just lost control and made a mistake	1.74	1.07	**.42**	.02	−.11	.02	.19	.31
5	Some children (14–16 years) behave in a seductive manner towards men	2.62	1.30	**.63**	.08	.14	.03	.47	.49
6	Sexual contact between a child (14–16 years) and a man causes the child no emotional problems	1.69	1.01	**.70**	−.01	−.08	.03	.49	.62
-	Sometimes children don’t say no to sexual contact with a man because they are curious about sex or enjoy it	2.24	1.17	**.62**	.07	.08	.06	.44	.54
7	Sometimes a child (14–16 years) instigates the sexual activities with a man	2.48	1.23	**.70**	.04	.14	.05	.55	.56
8	Some children (14–16 years) are eager and willing to have sexual contact with an adult	2.63	1.33	**.72**	.06	.17	−.01	.60	.55
9	Sexual contact between an adult man and a child (14–16 years) isn’t harmful for the child (physical and/or emotional)	1.55	0.87	**.76**	−.10	−.05	.03	.53	.66
-	Children who have been involved in sexual activities will eventually get over it	1.58	0.89	**.47**	.07	−.11	−.22	.29	.25
10	Only fondling the child (14–16 years) will not cause any harm (physical and/or emotional) to the child	1.47	0.79	**.60**	.04	−.24	−.09	.42	.36
11	Women are sly and manipulating when they want to seduce a man	2.59	1.21	.04	**.68**	.11	−.01	.49	.57
12	Women often falsely accuse men of rape	2.32	1.04	.07	**.68**	.02	−.07	.48	.61
-	It’s a dog-eat-dog world out there	3.17	1.17	−.06	**.47**	.14	.27	.35	.20
13	In general, women want sex, regardless of how they can get it	2.14	1.05	.04	**.49**	−.02	.11	.29	.38
14	Women often flirt with men just to tease or fool them	2.88	1.07	.01	**.49**	.13	.19	.34	.30
-	People should mostly look after themselves	3.20	1.20	−.02	**.43**	.07	.21	.26	.22
15	Often it appears a woman cares about a man, but in reality she is only out to use that man	2.00	1.04	.04	**.60**	−.04	.12	.43	.48
16	Normally, women can be trusted (R)	1.82	1.01	−.05	**.68**	−.27	−.04	.52	.41
17	It is safer not to trust women	1.74	0.98	−.05	**.64**	−.09	.08	.42	.55
18	Women often falsely accuse men of rape to protect their reputation	1.99	1.03	.05	**.69**	.07	−.07	.49	.62
19	Most women manipulate to get what they want	2.27	1.19	.13	**.69**	.10	.00	.57	.56
20	Most women do not lie to get ahead in life (R)	2.75	1.24	−.08	**.53**	−.14	−.06	.27	.39
21	Most men think about sex at least once a month	4.77	0.79	.10	.00	**.54**	−.06	.31	.44
22	Most men have read an erotic magazine or visited a porn website at least once in their life	4.58	1.00	.13	.01	**.53**	.05	.32	.40
23	Some men have had sexual fantasies about someone other than their partner while in a relationship	4.52	0.77	.19	.02	**.60**	−.07	.41	.41
24	Most men masturbate	4.61	0.80	.17	.09	**.56**	.04	.38	.39
-	Fondling a child (11 years or younger) without penetration can still cause harm (physical and/or emotional) (R)	1.70	1.26	.15	.18	**−.48**	−.11	.31	.30
25	Most things in life just happen to you	2.68	1.13	.30	−.03	−.01	**.54**	.38	.24
26	The world is full of danger	3.45	1.28	−.08	.27	.22	**.50**	.43	.23
27	People have no control over what happens to them in life	2.78	1.14	.05	.04	−.10	**.69**	.49	.59
28	Life is largely determined by random events	2.97	1.10	.11	.05	.01	**.60**	.40	.49
Coefficient Alpha				.89	.84	.69	.66		
Proportion of variance accounted for (%)				12	10	6	5		

*Note*. The extraction method was principal component analyses with an oblique rotation. Final components based on factor loadings (primary loading >.40; secundary loading <.30, difference primary - secundary loading >.20), parsimony and theoretical convergence are in bold. Results are based on the pattern matrix. Results from the structural matrix did not diverge based on the final model obtained. Only numbered items were included in the final scale. Possible answering ranging from 1 (disagree) to 5 (agree). h2 = comunnalities. Δ primary - secundary loading = difference between the primary and secundary loading. The presented items are translated with the utmost care, however, they do represent sample items translated to English. Reverse-scored items are denoted with an (R). Please see the online supplementary material for the full items.

### Measurement Invariance

Next, we tested measurement invariance in the general population and the (sub)clinical population sample using the previous established 28-item four-component solution. Full-information Maximum likelihood (FIML) was used to deal with missing values (0.3%). Before conducting MG-CFA, we first modeled the final four component model to the data containing both the general and (sub)clinical population, revealing approximate to good model fit, with S-Bχ^2^(*df*) = 605.816(338), *p* < .001; CFI = 0.940; RMSEA = 0.041; SRMR = .051, including high standardized factor loadings (all significant at *p* < .001).

The MG-CFA analyses testing configural invariance indicated that the same four factors and patterns of factor loadings exist in both the general and (sub)clinical population (Table 4). Constraining the factor loadings of the same factors to test for metric invariance did not worsen model fit. To test for scalar invariance, intercepts within the factors were constrained to be equal across groups, which did not worsen model fit, also not when more conservative thresholds were considered (i.e., ΔCFI ≤ .005; ΔRMSEA ≤ .010; Chen, 2007). Finally, to establish strict invariance, residual variances within the same factors were constrained and results suggested that strict invariance could not be obtained. When evaluating the criteria set before, the ΔCFA did not meet the criteria. The ΔS-Bχ^2^ test was significant when comparing scalar versus metric invariance and when comparing strict versus scalar measurement invariance. For the comparison between the scalar and metric model, however, the changes on the other set criteria (i.e., ΔCFI, ΔRMSEA, ΔSRMR) were below the specified threshold.

**Table 4.** Fit Statistics for Measurement Invariance of the Implicit Theories of Sexual Offense Questionnaire.

Model	S-Bχ2		Correction	ΔS-Bχ2	(Δ)*df*		*p*	CFI	ΔCFI	RMSEA	ΔRMSEA	SRMR	ΔSRMR
Model 1: Configural	1070.200	1.008				676		0.921		0.047		0.059	
Model 2: Metric	1100.753	1.010		30.553		24	0.150	0.919	−0.002	0.047	0.000	0.061	0.003
Model 3: Scalar	1150.594	1.008		49.841		24	<.05	0.914	−0.005	0.048	0.001	0.062	0.001
Model 4: Strict	1214.456	1.030		63.862		28	<.001	0.903	−0.011	0.050	0.002	0.064	0.002

*Note*. Analyses were conducted on the full sample (*N* = 496). The n for the general population = 427 and the n for the (sub)clinical population = 69. S-Bχ2 = Satorra-Bentler adjusted χ2 test statistic; ΔS-Bχ2 change in S-Bχ2; Correction refers to the scaling correction factor for the maximum likelihood robust (MLR) estimator. CFI = comparative fit index; ΔCFI = change in CFI; RMSEA = root mean-square error of approximation; ΔRMSEA = change in RMSEA; SRMR = standardized root mean-square residual; ΔSRMR = change in SRMR. All S-Bχ2 values were significant at p < .001.

To conclude, the four-factor model of the ITSOQ showed measurement invariance on the scalar level for the general and (sub)clinical population. These results indicate that possible mean differences between the general and (sub)clinical population on the ITSOQ scale scores indeed represent differences in the constructs being measured.

### Mean Differences

First, mean differences between the several population samples were calculated. Results indicate that the (sub)clinical population, based on Kruskal Wallis analyses, scored significantly lower on the SSDI, indicating a higher degree of sexual social desirability (see Table 5). Additionally, the (sub)clinical population sample scored significantly higher on the Uncontrollability factor compared to the general population. When comparing the (sub)clinical population, the sample population of men who violently offended scored significant lower (i.e., more sexual social desirability) on the SSDI than the general population sample, *U* = 2824.500, *p* = .01, *r* = −.14, AUC = .31. Additionally, the pedophilia-supportive forum users scored significantly higher on the Child factor compared to the population sample of men who sexually offended, *U* = 146.500, *p* = .03, *r* = −.13, AUC = .26. This effect disappeared however, when only the men who sexually offended against children (*M* = 2.12, *SD* = 1.18) were considered *H*(3) = 5.669, *p* = .129, ε2 = .01. Analysis including only the men who sexually offended against children can be found in the online supplemental material. It has to be noted that all effect sizes can be considered small (Cohen, 1992; for similarities between *r*; *ε2*, see Allen, 2017). In all analyses, we applied Holm’s correction (Holm, 1979) for multiple comparisons. Due to the small (sub)clinical population samples, these results should be interpreted with caution.

**Table 5.** Mean Differences Between the Different Population Samples of the Factors of the Implicit Theories of Sexual Offense Questionnaire.

	M (SD)			M (SD)			
				(sub)Clinical Sample			
	General Population Sample (*n* = 427)	(sub)Clinical Sample (*n* = 69)	Difference	Men Who Sexually Offended (*n* = 28)	Men Who Violently Offended (*n* = 21)	Pedophilia-supportive Forum Users (*n* = 20)	Difference
F1 Child	2.02 (0.77)	2.11 (1.07)	U = 15104, *p* = .712, r = −.02, AUC = .51	1.86 (1.07)	1.99 (0.91)	2.60 (1.13)	**H(3) = 9.805, p = .020, ε2 = .02**
F2 Women	2.25 (0.70)	2.4 (0.79)	U = 13012, *p* = .126, r = −.07, AUC = .44	2.33 (0.89)	2.65 (0.80)	2.24 (0.58)	H(3) = 5.767, *p* = .122, ε2 = .01
F3 SSDI	4.62 (0.62)	4.44 (0.61)	**U = 17922, p = .001, r = .14, AUC = .61**	4.38 (0.72)	4.36 (0.52)	4.61 (0.50)	**H(3) = 13.519, p = .004, ε2 = .03**
F4 Uncontr.	2.97 (0.82)	3.21 (0.84)	**U = 12410, p = .037, r =** **-.09, AUC = .42**	3.15 (1.00)	3.42 (0.68)	3.09 (0.73)	H(3) = 6.265, *p* = .100., ε2 = .01
IM	0.35 (0.23)	0.37 (0.23)	U = 14076, *p* = .449., r = −.03, AUC = .48	0.39 (0.21)	0.34 (0.19)	0.38 (0.28)	H(3) = 1.427, *p* = .699., ε2 = .00
SDE	0.28 (0.22)	0.30 (0.21)	U = 14076, *p* = .449., r = −.03, AUC = .48	0.34 (0.19)	0.34 (0.27)	0.20 (0.14)	H(3) = 6.5694 , *p* = .087., ε2 = .01

*Note*. While Kruskal Wallis analyses indicated significant group differences on F4 Uncontrollability, when comparing the specific population samples, no significant differences were found after applying Holm’s correction (Holm, 1979) for multiple comparisons. SSDI = Social Sexual Desirability Index. Uncontr. = Uncontrollability. IM = Impression Management. SDE = Self-Deceptive Enhancement. Significant values (p ≤ .05) are in bold.

### ITSOQ Factor Correlations, Social Desirability, and Sexual Interest

To quantify the degree to which the factors of the ITSOQ, social desirability scales, and the degree of self-reported sexual interest are related, Spearman correlational analyses were performed for the general and (sub)clinical population separately (see Tables 6 and 7). As samples sizes for the (sub)clinical population were too small to obtain stable effects (Schönbrodt & Perugini, 2013), direct comparisons with the general populations were not possible. Therefore, correlational results will be described for both populations separately.

**Table 6.** Correlational Analyses Between the Factors of the Implicit Theories of Sexual Offense Questionnaire and Social Desirability for the General and (sub)Clinical Population Samples.

	F1 Child	F2 Women	F3 SDDI	F4 Uncontr.	IM	SDE
F1 Child		.17	.19	.09	**−.28**	**−.27**
F2 Women	**.41**		−.09	**.38**	−.02	.01
F3 SDDI	**.25**	**.10**		.04	−.04	.01
F4 Uncontrolibility	**.18**	**.34**	**.14**		.20	−.18
IM	**−.23**	**−.29**	**−.13**	**−.12**		.22
SDE	**−.20**	−.03	.00	−.08	**.30**	

*Note*. Spearman Rho Correlation coefficients for the general population sample (*n* = 427) are presented under the diagonal; Correlation coefficients for the (sub)clinical population (*n* = 69) can be found above the diagonal. SSDI = Social Sexual Desirability Index. IM = Impression Management. SDE = Self-Deceptive Enhancement. Significant values (p ≤ .05) are in bold.

**Table 7.** Correlational Analyses Between Self-Reported Sexual Interest and the Factors of the Implicit Theories of Sexual Offense Questionnaire for the General and (sub)Clinical Population Samples.

	General Population								(sub)Clinical Population						
Sexual Interest Towards…	M (SD)	Range	% Sexual Interest	F1 Child	F2 Women	F3 SDDI	F4 Uncontr.		M (SD)	Range	% Sexual Interest	F1 Child	F2 Women	F3 SDDI	F4 Uncontr.
…adult women	84.70 (25.40)		0–100	96.47	.10	.08	**.21**	−.01	58.10 (41.60)	0–100	87.76	−.10	**.35**	.09	.08
...adult men	6.69 (19.60)		0–100	23.00	.09	**−.14**	.09	.00	16.60 (29.20)	0–97	38.78	**.29**	−.04	.09	−.03
...girls age 14–16	11.00 (19.60)		0–100	41.65	**.33**	.09	**.13**	.00	19.40 (32.20)	0–100	48.98	**.47**	.04	.20	−.23
...boys age 14–16	0.82 (4.86)		0–70	7.53	.05	−.07	−.01	−.04	19.60 (33.90)	0–100	38.78	**.43**	−.19	.17	−.13
...girls age 12–13	1.00 (3.72)		0–25	11.05					16.60 (29.00)	0–100	39.58	**.37**	−.03	.06	−.21
...boys age 12–13	0.32 (3.05)		0–58	4.93					21.30 (37.10)	0–100	36.73	**.37**	−.23	.12	−.11
...girls age ≤ 11	0.13 (0.85)		0–8	4.71					19.20 (35.30)	0–100	32.65	**.33**	−.11	.07	−.23
...boys age ≤ 11	0.39 (4.31)		0–81	4.70					22.10 (37.10)	0–100	34.69	**.37**	−.24	.17	−.09

*Note*. Spearman Rho analyses. For the general population sample it was not possible to look at the correlation between sexual interest with boys age 14–16, sexual interest towards girls/boys age 12–13, sexual interest towards girls/boys age ≤ 11 and the factors of the ITSO due to a general low score on these items. % sexual interest indicates any indication (% > 0) on the item. SSDI = Social Sexual Desirability Index. Uncontr. = Uncontrollabiliy. The n for the general population = 424/425. The n for the (sub)clinical population is lower due to the fact that men who sexually offended from one inpatient clinic did not answer the questions concerning interest. The n for the (sub)clinical population = 48. Significant values (p ≤ .05) are in bold.

#### General Population

In the general population, all factors had small to medium positive correlations with each other. Regarding social desirable responding, the same trend for the SSDI and IM was found. That is, a small positive correlation with the other factors of the ITSOQ and the SSDI was found, indicating that in the general population, less desirable responding on the SSDI (i.e., higher scores on the SSDI indicate less social desirable responding) was associated with higher scores on the clinical scales. With regard to the correlation of the ITSOQ factors with the IM and SDE scales of the BIDR-D20, significant small negative correlations between IM and the ITSOQ factors were found. For SDE, small negative correlations with the Child factor was found, indicating that more desirable responding was associated with lower scores on the child scale.

With regard to self-reported degree of sexual interest, significant medium and small positive correlations were found between a higher degree of self-reported sexual interest towards girls between the age of 14–16 years and higher scores on the Child factor of the ITSOQ. Association with the ITSOQ factors and sexual association towards the other age categories or towards boys could not be determined due to the generally low degree of sexual interest on average. Additionally, more self-reported sexual interest in minors was significantly negatively associated with scores on the women factor (albeit with a small effect). Lastly, a small positive significant association was found between the SSDI factor and a higher degree of sexual interest in adult women and girls between 14-16 years. Controlling for SSDI, IM or SDE did not affect the results.

#### (sub)Clinical Population

With regard to the correlation between the ITSOQ factors, only a significant medium correlation was found for the Women and the Uncontrollability factor. Considering social desirable responding, higher scores on IM and SDE were significantly associated (small effect) with lower scores on the Child factor.

Regarding the self-reported degree of sexual interest and the factors of the ITSOQ, a higher degree of self-reported sexual interest in a minor (14–16, 12–13 and ≤ 11 years) was significantly associated with higher scores on the Child factor, with medium effect sizes. Self-reporting a higher degree of sexual interest in adult women was found to have a significantly positive medium association with higher levels on the Women factor. Controlling for either SSDI, IM or SDE did not affect the results. See the online supplemental material for the partial correlation analyses for both the general and (sub)clinical population.

## Discussion

The current study was designed to develop a questionnaire to assess the seven core ITs of sexual offending as described earlier by Ward and colleagues (Polaschek & Ward, 2002; Ward & Keenan, 1999). We used existing cognition questionnaires to create an item pool. Items were selected based on descriptive statistics (e.g., mean, standard deviation, range). Subsequently, PCA identified a four-component solution with three factors reflecting IT content and one factor reflecting a sexual social desirability index. Of the three factors reflecting IT content, two components were victim-specific ITs (children; women), and one component resembled the antisocial uncontrollability IT. Invariance analyses indicated that mean differences between the general and (sub)clinical population on the ITSOQ reflected actual group differences. No significant differences between the different population samples were found on the two victim-specific factors. However, the (sub)clinical sample showed higher scores on the antisocial uncontrollability factor and lower SSDI scores (i.e., indicating more social desirable responding) compared to the general population sample. Effect sizes were generally low to moderate. Additionally, albeit small, associations between self-reported sexual interest in children and adults and the victim-specific child and women components were found, respectively. Detailed discussion of the findings follows in subsequent sections.

### Victim-Specific ITs

As predicted, victim-specific ITs grouped together based on specific victim content. That is, the child-specific content, which could be argued to be part of Wards’ *Child as sexual beings* and *Nature of harm* ITs, resulted in one factor. Furthermore, content related to Wards’ *Women are dangerous* and, to a lesser extent, *Women as sex objects* ITs grouped together in a women-specific factor. Both factors had satisfactory to good reliability (Bland & Altman, 1997). In addition, as hypothesized and in line with previous research (Ó Ciardha, 2011; Ward & Beech, 2006), more self-proclaimed sexual interest in minors was associated with higher expression of IT content.

As recommended by the men residing in the forensic mental health system during the pilot phase, items referring to a child were reformulated using two different age categories: 11 years and younger and 14–16 years old. The Child factor resulting from the analyses, however, only included items referring to children of 14–16 years. When looking at the initial item pool, results indicated that the questions referring to children of 11 years and younger were not answered affirmingly (i.e., mean scoring of at least 2) in most of the population samples. While some items referring to this specific age category yielded some scores in the pedophilia-supportive forum users, mean scores leaned towards the lower end of the scale. Additionally, all samples showed higher scores on items referring to 14–16 years compared to 11 years and younger. While the forensic mental health system clients indicated during the pilot phase of the study that they answered conservatively due to the lack of an age specification, perhaps specifying the age in the questions highlighted the possible differences in answering between the categories, resulting in lower scores on the items referring to younger children.

### General Antisocial ITs

The general antisocial IT concerning uncontrollability tapped into a sort of helplessness in life. Within this factor, three items addressed uncontrollability in life events, whereas one item referred to the perception of danger in life. Although the danger item might be more relevant for the *Dangerous world* IT, we argue that the items in this IT also capture uncontrollability in the sense of helplessness (i.e., “People have no control over what happens to them in life”). The same uncontrollability was reflected in the other three items.

No evidence was found for a separate factor in the ITSOQ that taps into Ward’s *Entitlement* and *Dangerous world* ITs. As proposed by Gannon (e.g., Gannon et al., 2008), we used general statements as they would better reflect IT content. However, in hindsight, one could question the suitability of general statements for ITs related to entitlement and a dangerous world. That is, whereas most ITs can be seen as being outside the person’s own context (children, women), entitlement for instance can be viewed as something that only revolves around the person who holds the IT. Entitlement can be defined as “a stable and pervasive sense that one deserves more and is entitled to more than others” (Campbell et al., 2004, p. 31), making it a concept that only holds for the person itself, and less for men in general.

### Social Desirability

Besides the factors that indicated possible IT content, we also identified a factor including items related to sexual social desirability. This factor (the SSDI) comprised sexual behaviors typically associated with men that are often kept private due to social norms (i.e., masturbation, viewing porn websites). The items of the SSDI were also formulated as general statements, thus reflecting general social norms rather than individual self-reflections (e.g., “Some men have had sexual fantasies about someone other than their partner while in a relationship”), and hence may not capture the essence of social desirability. However, given the inherent sensitivity of cognitions surrounding sexual (offending) behavior and the “ground truth” that cannot be established, the SSDI could be an indicator of response patterns. Controlling for it could reduce the tendency for socially desirable responding.

Regarding the possible influence of social desirability on the ITSOQ, results indicated that for the SSDI and the BIDR-D20 IM scale, lower scores on the ITSOQ factors were associated with a higher level of social desirability for the general population. For the BIDR-D20 SDE, this pattern was only found for the Child factor. For the (sub)clinical population, only the Child factor displayed small associations: Higher scores on both IM and SDE were associated with lower scores on the Child factor. These results suggest that the ITSOQ is partly influenced by social desirable responding. That said, controlling for social desirability using either the SSDI, IM or SDE scales, did not affect the associations between sexual interest and the ITSOQ factors.

### Base Rate of Affirmation

In line with other studies (e.g., Merdian et al., 2014), base rates of item endorsement were generally low on all items for both the general and (sub)clinical sample, except for the SSDI (as expected). One reason for the low base rate in the (sub)clinical population sample may be the low level of anonymity in clinical settings, or that treatment affects answers (e.g., Benbouriche et al., 2015). However, the inclusion of the pedophilia-supportive forum users renders these arguments less plausible in the current study. The pedophilia-supportive forum users participated anonymously: an untraceable link was used when collecting the data and this was made clear to the participants.

Based on the non-significant differences between the men residing in the forensic mental health system and pedophilia-supportive forum users, combined with the low rate of affirmation, it appears that anonymity may not have significantly influenced responses. Additionally, since the forum users were not in (mandatory) treatment during the assessment, this does not account for the low rate of general agreement on the statements. It could be argued that the forum users represented a subgroup with pedophilic feelings that recognize the consequences of acting on these feelings, and thereby did not identify with the statements used. Perhaps, in the context of sexual and antisocial cognitions, one should not seek to average out answers to a wide variety of statements as is normally done with questionnaires, but instead select items in which the percentages of participants’ affirmation is relatively high.

A reason why the seven proposed ITs are frequently found in interview studies (e.g., Keown et al., 2010), and not with the same frequency in studies using questionnaires, may be attributed to the comprehensive discussion of ITs in interviews. Interviews often explore ITs within a broader framework containing several aspects, a depth that is difficult to replicate in questionnaire items. Additionally, a self-report can never account for the heterogeneity of the population of men who sexually offended and tailor questions accordingly.

Alternatively, some researchers argue that the presence of ITs during interviews may stem from the natural course of conversation, and in which the offense committed often is a topic of discussion. This engagement could potentially prime the relevant cognitive structures, leading to schema activation (e.g., Gregg et al., 2006). Moreover, interviews may elicit more situation-specific justifications rather than enduring beliefs when it comes to the offense. In light of this, the question remains whether these ITs are merely theoretical constructs, or whether it is feasible to empirically substantiate their existence as networks of interrelated beliefs that people use to interpret (interpersonal) situations (e.g., by rejecting or reinterpreting evidence conflicting with the IT), and direct goals, desires, and behaviors in general.

### Limitations

Several limitations should be acknowledged when interpreting the findings. The first limitation arises from the relatively small (sub)clinical samples, especially of men who sexually offended against adult women. Though common in forensic research to include small(er) samples (e.g., Paquette & Cortoni, 2019), it does pose problems. For example, although we found measurement invariance for general and (sub)clinical samples, combining population samples for such analyses is suboptimal. Additionally, the different (sub)clinical samples lacked power to identify medium to small differences between the groups. Due to the low number of (sub)clinical samples, most of the analyses were done on the general population. It is possible that a different pattern of results would have been obtained if the samples sizes were reversed (i.e., 400 persons with a sexual offence history and 70 men from the community). It should be noted that measurement invariance analyses indicated that any mean differences between the general and (sub)clinical population on the ITSOQ scale scores indeed represent differences in the constructs being measured.

Additionally, the use of a neutral answer option is considered by some as a limitation. While the choice for a neutral option in this study was a deliberate choice, its use is debated, as selecting it by the respondents may convey indifference (e.g., Johns, 2005) and potentially lead individuals to claim to have no opinion when they actually do hold one (e.g., Krosnick et al., 2002).

Lastly, although the use of existing questionnaires to develop a new self-report measure is a recommended technique and we asked participants in the pilot phase whether they missed certain information, this approach may have overlooked valuable insights or unique perspectives. While we asked during the pilot phase whether we missed certain information and questions, we could have extended this to staff and other experts in the field. In addition, a more elaborate interview could perhaps have given more information.

### Future Research

Future work could focus on further validating the ITSOQ using a large (sub)clinical sample. Reframing the items in the more general antisocial ITs (*Entitlement*, *Dangerous world*) to reflect statements indicating the person’s own perspective compared to the general statements would be of interest. One could also examine if existing questionnaires that measure entitlement, hostility or grievance would fit the description given by the original authors of the general antisocial ITs (Polaschek & Ward, 2002; Ward, 2000; Ward & Keenan, 1999). For example, for the IT *Dangerous world* subscales of the World Assumption Scale (WAR; Janoff-Bulman, 1989) could be used. The WAR measures fundamental schemas, embedded within a person’s conceptual system regarding their assumptions of the world around them, such as the assumption that other people are basically good, kind, helpful, caring, and trustworthy (Janoff-Bulman, 1989). Such questionnaires or scales may resemble antisocial ITs better than the items used in the current study.

To avoid low base rates of affirmation, further research could investigate whether the current Likert scales are suitable for the assessment of ITs. That is, it could be explored what the effect is of using different scaling options. On the one hand, labeling only end points may introduce a bias toward extreme answers (Weijters et al., 2010). On the other hand, research indicated that labeling all points increases positivity bias (Krosnick, 1991). The latter outcome, however, likely not materialize in the current study. Additionally, increasing the answering options could be explored as it is found that this decreases the occurrence of response styles (Weijters et al., 2010), and increases reliability (Lozano et al., 2008). Besides looking at the scaling options, several other scoring options could be investigated, such as a more dichotomous scoring approach In that case, it could be analyzed whether participants (either general population of (sub)clinical) agree or strongly agree on these items.

To assess cognitions in men who offended, the use of indirect measures is often suggested as a direction for future research (e.g., Babchinshin et al., 2013). We are aware of the criticism of the use of self-report assessment of ITs and the change in research direction towards latency-based indirect measures (i.e., measures to assess constructs under automaticity). However, at present, self-reports can still be considered useful to assess ITs of men who sexually offended in clinical practice. They are cost-effective, easy to administer and interpret, and do not require extensive training.

### Practical and Societal Relevance

ITs of men who sexually offended are often referred to as a general world view concerning children, women and antisocial aspects that is applicable throughout someone’s thinking, goals, and acting in daily life. Therefore, the assessment of these ITs should go beyond the information concerning the offense committed. That is, regarding Mann and Beech’s (2003) schema based model of sexual offenses, they state that challenging offense-specific cognitions would not address the problem, because this (also) arises from distortions in a general world view. In light of this, we propose that the factors of the ITSOQ could serve as a starting point of conversation concerning an individual’s perspective on their own (possible) victims and situation. The ITSOQ could aid in distinguishing between a person’s situation-specific cognitions and domain-general, core beliefs. Additionally, the findings from the ITSOQ could be linked to the maladaptive schemas described by Young (1990). For instance, Fisher and Beech (2007) argue that uncontrollability would align with Young’s schema “insufficient self-control/self-discipline”, indicating an inability or unwillingness to use adequate self-control or frustration tolerance, thus failing to regulate the expression of emotions and impulses (Young et al., 2006).

The inconclusive results regarding the effect of socially desirable responding on the ITSOQ and the ongoing discussion concerning socially desirable responding warrant caution. Although socially desirable responding is relevant in (clinical) assessment, the questions of how to handle socially desirable responding, or what it reflects, are open for discussion. Some researchers believe that socially desirable responding is a short-term response influenced by situational factors (e.g., Edwards, 1957; Nederhof, 1985), while others see it as a more lasting trait (e.g., Paulhus, 2002) that may reflect an individual’s ability to adapt to social situations and seek approval from others. While some researchers advocate for adjusting individual scores based on social desirability scores (e.g., van de Mortel, 2008), others suggest that this adjustment would remove important variability (e.g., Uziel, 2010). We suggest using the SSDI to indicate response patterns, as higher scores are expected on these items. If (none of) the items of the SSDI are scored affirmatively, one could, therefore question the truthfulness of the responses on IT items.

### Conclusion

To conclude, we believe that the ITSOQ could be an addition to the clinical and research field on sexual offending in given a direction towards pertaining ITs. Future research could investigate if the ITSOQ is a valuable tool in determining the discrepancy between the presence of ITs (networks of interrelated beliefs that people use to understand and explain their social world) and cognitions presented only in the context of the offense(s). Further research regarding the validity, the age specification and possible antisocial IT scales is warranted to further develop and validate the measure.

## Supplemental Material

Supplemental Material - Construction and Validation of the Implicit Theories of Sexual Offense Questionnaire (ITSOQ) in a General and (sub)Clinical Population Sample Supplemental Material for Construction and Validation of the Implicit Theories of Sexual Offense Questionnaire (ITSOQ) in a General and (sub)Clinical Population Sample by Mirthe G. C. Noteborn, Martin Hildebrand, Jelle J. Sijtsema, Jaap J. A. Denissen, and Stefan Bogaerts in Sexual Abuse

## Notes

1. On request of the reviewer we also included the AUC as an effect size based on the formula U/(*n*1 * *n*2).

2. We are aware of the different labels used for forum users and the discussion on the nature and intent of this forum. As this paper is intended for the purpose of questionnaire development and out of respect for the forum moderators, allowing participation under conditions of mutual respect, we decided not to use specific labels (see Jahnke et al., 2022). We tried to describe the forum users as best as possible without judgement or stigmatization, focusing on a descriptive rather than an evaluative approach.

3. Questionnaires to assess cognitive distortions of men who sexually offended against children were*:* The MOLEST scale (Bumby, 1996); the Abel and Becker Cognition Scale (ABCS; Abel et al., 1989); the Hanson Sex Attitude Questionnaire (HSAQ; Hanson et al., 1994); the Cognitive Distortions and Immaturity (CDI) scale of the Multiphasic Sex Inventory (MSI; Nichols & Molinder, 1984); the Offenses Against Children (OAC) scale of the Questionnaire on Attitudes Consistent with Sexual Offending (QACSO; Lindsay et al., 2007); the Sex with Children (SWCH) scale (Mann et al., 2007); the Children and Sexual Activities (C&SA) scale (Howitt & Sheldon, 2007). Questionnaires to assess cognitive distortions of men who sexually offended against adult women were*:* Burt’s (1980) Rape Myth Acceptance (RMA), Adversarial Sexual Beliefs (ASB), and Acceptance of Interpersonal Violence (AIV) scales; the Hostility Towards Women (HTW) scale (Check et al., 1985); the Attitudes Towards Women Scale (AWS; Spence et al., 1973); the Bumby RAPE scale (Bumby, 1996); the Rape and Attitudes to Woman (RAW) scale from the QACSO (Lindsay et al., 2007).
