# Supplementary material for: Construction and Validation of the Implicit Theories of Sexual Offense Questionnaire (ITSOQ) in a General and (sub)Clinical Population Sample
Source: Sex Abuse. 2025 Apr 10;37(8):910–49. doi: 10.1177/10790632251326555 (PMC12552757; doi:10.1177/10790632251326555)
Supplement: Supplemental Material - Construction and Validation of the Implicit Theories of Sexual Offense Questionnaire (ITSOQ) in a General and (sub)Clinical Population Sample [file sj-pdf-1-sax-10.1177_10790632251326555.pdf]

Guide to the Online Supplementary Material

"Construction and Validation of the Implicit Theories of Sexual Offense Questionnaire (ITSOQ) in a General and (sub)Clinical Population Sample"

The online supplementary material is meant to supplement the material presented in the full paper. The goal of this information is to supply interested readers with more details surrounding the results presented in the full paper. Below, the sections included in the online supplementary material are described.

1. Development of the Implicit Theories of Sexual Offense Questionnaire (ITSOQ)

This file is supplementary to the main text in the full paper's method section and it contains a more elaborate account of the development of the ITSOQ.

2. Item Reduction and Scoring Differences in Age

This file is supplementary to the main text in the result section named item reduction. Online supplementary material 2 contains the initial item pool of the ITSOQ and the descriptives of the items in the initial item pool (Mean, SD, Range, Skewness, Kurtosis). Additionally, tables with the Wilcoxon signed-rank test looking at the difference between the items with the different age categories (≤ 11 years versus 14-16 years) is included complementary to the main text in the analysis section of the manuscript.

3. Principle Component Analysis

Online supplementary material 3 is complementary to the Principle Component Analysis in the result section of the full paper. The file contains the items included in the PCA, the results of the PCA for the 4-, 5- and 6-component solutions including the pattern and structure matrix.

4. Mean Differences Men Who Sexually Offended Against Children

online supplementary material 4 is complementary to the result section in the full paper. It contains the mean differences between the several population samples with the sample of men who sexually offended only including men who sexually offended against children.

5. Sexual Interest and the ITSOQ, controlled for Social Desirability

Online supplementary material 5 is complementary to the results section in the full paper. It contains the correlational analyses between sexual interest and the factors of ITSOQ for the general and (sub)clinical population samples controlled for social desirability.

All files are meant to be helpful for properly interpreting the output, understand and evaluate the conclusions made in the full paper as well as making our results reproducible.

3.1. Development of the Implicit Theories of Sexual Offense Questionnaire (ITSOQ).

This file is supplementary to the main text in the full paper's method section and it contains a more elaborate account of the development of the ITSOQ.

Development of the Implicit Theory of Sexual Offense Questionnaire (ITSOQ)

The development of the ITSOQ consisted of five steps and a pilot, which are outlined below.

Step 1

In Step one, three of the authors independently mapped items from existing cognition questionnaires into the respective implicit theories (ITs) (i.e., *Entitlement*, *Uncontrollability, Dangerous world*, *Children as sexual beings, Nature of harm*, *Women as sexual objects*, *Women are dangerous*) developed by Ward and colleagues (Polaschek & Ward, 2002; Ward & Keenan, 1999;). Below, the (subscales of) questionnaires used to identify potential relevant items for the ITSOQ are described (*N* = 314 items).

Questionnaires Used to Assess Cognitive Distortions of Men who Sexually Offend against Children).

The MOLEST scale (Bumby, 1996); the Abel and Becker Cognition Scale (ABCS; Abel et al., 1989); the Hanson Sex Attitude Questionnaire (HSAQ; Hanson et al., 1994); the Cognitive Distortions and Immaturity (CDI) scale of the Multiphasic Sex Inventory (MSI; Nichols & Molinder, 1984); the Offenses Against Children (OAC) scale of the Questionnaire on Attitudes Consistent with Sexual Offending (QACSO; Lindsay et al., 2007); the Sex with Children (SWCH) scale (Mann al., 2007); and the Children and Sexual Activities (C&SA) scale (Howitt & Sheldon, 2007) were used.

Questionnaires to Assess Cognitive Distortions of Men who Sexually Offend against Adult Women.

We used Burt's (1980) Rape Myth Acceptance (RMA), Adversarial Sexual Beliefs (ASB), and Acceptance of Interpersonal Violence (AIV) scales; the Hostility Towards Women (HTW) scale (Check et al., 1985); the Attitudes Towards Women Scale (AWS; Spence et al., 1973); the Bumby RAPE scale (Bumby, 1996); the Rape and Attitudes to Woman (RAW) scale from the QACSO.

Of the total of 314 items, 35 items (17 to assess cognitions of men who sexually offended against children, and 18 to assess cognitions of men who sexually offended against adult women) were randomly selected in order to examine the interrater reliability between the three raters. At least two items from each questionnaire were included. Each of the three raters categorized the 35 in the respective IT. If an item did not fit any of the respective ITs, according to the raters, the item was coded as 'Other' (i.e., item does not fit one of the respective ITs). With regard to the agreement between the three raters, absolute agreement was reached for 25 of the 35 items (71%) (*Entitlement* = 8; *Uncontrollability* = 4; *Dangerous world* = 3; *Children as sexual beings* = 4; *Nature of harm* = 2; *Women are dangerous* = 3; *Other* = 1). An interrater reliability analysis using Fleiss' kappa statistic, κ (Fleiss, 1971; Fleiss et al., 2003) was performed to determine consistency among raters, $\kappa = .74$, $p < .001$, 95% Cl [.66, .82]. Reliability between pairs of raters ranged from $\kappa = .66$, $p < .001$, 95% Cl [.52, .80] to .86, $p < .001$, 95% Cl [.72, 1.00], indicating adequate to excellent reliability (Landis & Koch, 1977). Next, the three raters independently categorized the remaining 279 items of the selected questionnaires based on IT. Absolute agreement between the three raters was reached for 157 of the 279 items (56%). Interrater reliability for all three raters was $\kappa = .64$, $p < .001$, 95% Cl [.61, .67], indicating appropriate reliability. For rater pairs, interrater reliability ranged from .52, $p < .001$, 95% Cl [.47, .57] to .83, $p < .001$, 95% Cl [.79, .88]. Interrater reliability for items that assessed distortions of men who sexually offended against children or against adult women, based on question content or target population of the questionnaire, was approximately the same (sexual offending against $\text{children}_{\text{content}}$ $\kappa = .62$, $p < .001$, 95% Cl [.57, .67] vs. sexual offending against $\text{children}_{\text{targetpopulation}}$ $\kappa = .64$, $p < .001$, 95% Cl [.60, .68]; sexal offending against adult $\text{women}_{\text{content}}$ $\kappa = .55$,$p < .001$, 95% Cl [.50, .61] vs. sexual offending against $\text{women}_{\text{targetpopulation}}$ $\kappa = .55$, $p < . 001$, 95% Cl [.49, .61]). All interrater reliabilities - bearing in mind the $\kappa = .49$ lower bound - were considered to be

moderate to excellent (Landis & Koch, 1977). Overall interrater reliability of all items (*N* = 314) was considered appropriate, $\kappa = .65$, $p < .001$, 95% Cl [.62, .68].

Of the 314 items, 182 items were selected based on agreement between the three raters; 158 were classified as belonging to an IT and 24 items were categorized by all raters as not belonging to any IT. Based on the content of the question, the 158 items classified as belonging to an IT were distributed among the different ITs as follows (see also Figure 1): *Entitlement* 29 items (Men who sexually offend against children = 6; men who sexually offend against adult women = 7; general antisocial = 13; victim unspecific = 3); *Uncontrollability* 22 items (men who sexually offend against children = 9; men who sexually offend against adult women = 7; victim unspecific = 6); *Dangerous world* eight items (Men who sexually offend against children = 8; men who sexually offend against adult women = 0); *Children as sexual beings* 50 items; *Nature of harm* 25 items; *Women as sexual objects* 15 items; *Women are dangerous* nine items. Considering the questionnaires that assess cognitions of men who sexually offended against children only, the proportions of these numbers are in line with the study of Gannon et al. (2008), indicating an underrepresentation of statements regarding nonsexual offense-specific cognitions in these questionnaires (i.e., the *Uncontrollability*, *Entitlement*, and *Dangerous world* ITs). That is, higher number of items related to the three general antisocial ITs were found in the current study, however, mostly found in the questionnaires concerning men who sexually offend against adult women, which were not included in the study of Gannon et al. (2008).

Figure 1

Overview of the Items with Absolute Agreement.

	Absolute Agreement	Original Articles	Total
Entitlement			
Men who sexually offend against children	6	2	8
Men who sexually offend against adult women	7	7	14
General antisocial	13	1	14
Victim unspecific	3	2	5
Uncontrollability			
Men who sexually offend against children	9	0	9
Men who sexually offend against adult women	7	7	14
General antisocial	0	0	0
Victim unspecific	6	8	14
Dangerous world			
Men who sexually offend against children	8	4	12
Men who sexually offend against adult women	0	0	0
General antisocial	0	5	5
Victim unspecific	0	4	4
Child as sexual beings	50	11	61
Nature of harm	25	7	32
Women as sexual objects	15	8	23
Women are unknowable	9	4	13

Due to the lack of items reflecting a general antisocial *Uncontrollability* and *Dangerous world* in general, the literature was searched for possible statements of these ITs indicated by the original authors (Polaschek & Ward, 2002; Ward & Keenan, 1999;), which resulted in an additional 70 items, with 12 items representing *Entitlement*, 15 items covering *Uncontrollability*, and 13 items for the *Dangerous world*.

Step 2

In Step two, it was checked if the ITs were represented by the selected items. For example, the *Dangerous world* IT is about (1) the belief that the world is hostile and people behave in an abusive and rejecting manner, and (2) the belief that adults are unreliable and children are dependable. Therefore, this IT must contain items (statements) that assess the belief that the world is a hostile place, items reflecting the belief that adults are unreliable and children are dependable. After examination of the items, it was concluded that all aspects of the respective ITs were represented. Also, 61 content-identical items were identified by two

of the auteurs in agreement. Furthermore, because the ITSOQ is intended to assess ITs of men who sexually offended against children or women, victim unspecific items (23 items) are unfavorable. Therefore, to increase the number of offense-specific items, the victim unspecific items were transformed in a way that they characterized one of the two offense categories. For example, the victim unspecific item “She is flirting and teasing me, so she wants to do it” was changed in the offense-specific item “If a child is flirting and teasing, it wants to have sex”).

Step 3

In step 3, a selection was made based on the presence of all aspects of the ITs, formulation of the question (e.g., double barreled items were deleted due to possible bias) and similar content as identified in step 2. This resulted in a total of 114 items.

Step 4

In step 4, the 114 items were translated into Dutch and reformulated in general statements as this is considered the best way to tap into IT constructs (Gannon & Polaschek, 2006; Gannon et al., 2009). In order to eliminate possible biases in the formulation of the items (for an overview, see Choi & Pak, 2005), all items were reviewed by three authors using a questionnaire appraisal system (QAS; Willis & Lessler, 1999). The QAS allows questionnaire designers to identify potential problems in the items’ wording or structure by evaluating step by step if the item exhibits features (e.g., technical terms, vague items, undefined and unclear common terms, double-barreled items) that are likely to cause problems. Questions were adjusted accordingly. Also, in step four, a five-point Likert-type scale was developed, with categories ranging from 1 (*disagree)* to 5 (*agree)* with the midpoint indicating a neutral position. Where with specific topics, using a five-point scale may not be the best choice as a neutral option (the midpoint) does not indicate a preference, we believe that in the case of sensitive topics, a neutral answer option might be considered a

more ‘safe’ option for participants harboring cognitions, while at the same time this answer option can be considered deviant from the norm (e.g., being neutral to sexual contact with a minor). Finally, feedback was provided by several professionals from different fields. One item was removed based on the resemblance with other items; this resulted in a total of 113 items.

Step 5

In Step 5, we developed a social desirability scale that focused on sexual content, which we named the Sexual Social Desirability Index (SSDI; e.g., “Most men masturbate”). The SSDI consists of seven items with sexual content based on “social rules” that would be expected to be answered conservatively. While it is acknowledged that general statements are less socially sensitive than personalized statements, formulating the questions differently could be distracting and affect the response on other statements. For this reason, one could not automatically assume that the developed SSDI items capture the essence of social desirability. However, as research has indicated that offense-supportive beliefs are socially sensitive and the “ground truth” cannot be established, these SSDI questions could indicate response patterns. Furthermore, these are all statements that can be assumed that men would generally answer affirmably. Including the seven social desirability questions in the item pool resulted in a total of 120 items.

Step 6

Step 6 consisted of a pilot. To see if differences in formulation and specificity of the items affected answering responses during the pilot phase, seven items were added twice in the item pool. Of these seven items, four were included using the term abuse/offense, and using the term sexual contact (i.e., “If a person does not use force to have sexual contact with a child, it will not harm the child as much” vs. “If a person does not use force when sexually abusing a child, it will not harm the child as much”). Furthermore, three items were added

indicating a difference in the age specification, with 13 years and younger as a category ("A child of 13 or younger can make their own decisions as to whether she (he) wants to have sex with an adult or not" vs. "A child can make their own decisions as to whether to have sexual activities with an adult or not").

Ten men who sexually offended, involuntary admitted to a forensic psychiatric institution in the Netherlands, were asked to fill out the 120-item preliminary version of the ITSOQ and provide us with detailed feedback. The 10 participants were handed an information letter containing detailed information about the procedure, duration, anonymity concerning scientific research and towards treatment staff, medical ethical approval, and that possible feelings of emotional discomfort could occur due to the content of the questions. Six men decided to participate.

Characteristics of the Men who Sexually Offended based on Index Offense

Two men had an index offense against a child, one against adult women, and three of the men who resided in forensic inpatient facilities offended against children as well as adult women. The youngest victim was three years old. Five participants offended against an extrafamilial victim, one against an intrafamilial victim. Two men had only contact index offenses, and four committed both contact and non-contact offenses. Five men who sexually offended had female victims, one had a male victim.

Characteristics of the Men who Sexually Offended based on Index Offense and Offense History

Regarding offense history, four participants can be classified as generalists, and two as specialists. Two men only committed contact offenses, while four committed contact as well as non-contact offenses. Four participants had only extra-familiar victims, and two had extra-familiar and intra-familiar victims (stepchildren). Two offended only against children, one against only adults, and three against both.

Feedback

The main feedback of the participants concerned the lack of an age specification regarding children and sexual contact in the items in which age could influence the scoring. Respondents indicated that they answered conservatively on these questions as they recognized that, for instance, a child of seven years old does not have the cognitive capacity to decide whether or not to have sexual contact. Regarding the items that were added and contained an age differentiation, participants did not seem to differ in their scoring. When asked what an appropriate age differentiation should be, participants indicated a cut-off of 14 years. For this reason, items in which the age of the child could influence the response (e.g., “If a child flirts with men, this means that the child wants to have sexual contact”) were asked separately for children between 14-16 years versus 11 years and younger. This age differentiation was applied to eliminate a possible grey area of sexual maturation (i.e., 12-13 years). After taking a critical look at the questions, only the age category 11 years and younger was used for some items, as using an older age category would be questionable (e.g., age category of 14-16 in the item “A child that walks around naked is indicating sexual contact”).

Additionally, some of the participants were under the impression that the questions needed to be answered based on “facts” as they were phrased as general statements (e.g., “I have seen this on TV”; “I have heard some men in the group say these things”). Therefore, the instructions were adjusted to ensure that the participants understood that the items needed to be answered according to their views.

When comparing items on the use of the phrase (wording) sexual contact vs. sexual abuse, it was found that the scoring of these items did not differ between the use of contact vs. abuse. However, when asked about the difference the participants indicated that in fact they thought there was a difference and that not all sexual contact is abuse. Therefore, the

terminology of the respective items was changed to sexual contact. Additionally, based on suggestions of the participants, several minor changes were made in some of the questions (e.g., items were difficult to read, participants did not know the meaning of a word). The final item pool comprised 131 items (See online supplementary material 2).

2. Item Reduction and Scoring Differences in Age

This file is supplementary to the main text in the result section named item reduction. Online supplementary material 2 contains the initial item pool of the ITSOQ and the descriptives of the items in the initial item pool (Mean, SD, Range, Skewness, Kurtosis). Additionally, tables with the Wilcoxon signed-rank test looking at the difference between the items with the different age categories (≤ 11 years versus 14-16 years) is included complementary to the main text in the analysis section of the manuscript.

Table 1

Initial Item Pool of the Implicit Theories of Sexual Offense Questionnaire (ITSOQ)

nr.	Items
1	If someone's child (stepchild or family member) does what is asked sexually, this means that the child also wants it
2	Society makes a much bigger deal out of sexual activities with children than it really is
3	It is okay if an adult male engages in sexual activities with children as a way of getting back at someone, for example the partner, a parent, or
4	If a woman does nothing to prevent herself from being raped , she doesn't mind it
5	Women have the right to make their own decisions regarding sexual contact (R)
6	Women are sly and manipulating when they want to seduce a man
7	Fondling a child can be considered as being sexual with a child (R)
8	Children (14 to 16 years) who have been involved in sexual activities with an adult male, will eventually get over it and get on with their lives
9	Most men think about sex at least once a month
10	It is OK to have sexual contact with a child (14 to 16 years) as long as the child is not forced into it
11	Some children (11 years or younger) are eager or willing to have sexual contact with an adult
12	Women often falsely accuse men of rape
13	Some children (14 to 16 years) are mature enough to enjoy sexual activities with men
14	Men rarely become suspicious of women who are friendlier than they expect
15	Rape is generally the results of misinterpreted cues
16	If a woman goes home with a man on the first date, she probably wants to have sex with him
17	It is society's reaction, rather than the sexual activities itself, which causes the distress a child feels
18	Many people are out to hurt each other
19	A child (14 to 16 years) can make its own decisions as to whether or not to have sexual contact with a man
20	If a woman kisses a man and she lets things get out of hand, it is her own fault if the man forces her to have sex with him
21	Some men are not "true" child molesters - they have just lost control and made a mistake
22	Most things in life just happen to you
23	Most men have read an erotic magazine or visited a porn website at least once in their life
24	Some men turn to sexual contact with children because they are deprived of sex with their adult partner
25	Sexual contact between an adult and a child (11 years or younger) isn't harmful for the child (physical and/or emotional)
26	In most cases when a woman is raped, she was asking for it
27	Children are more accepting and loving than adults are
28	Women are there to meet men's sexual needs regardless of their own needs
29	Children are supposed to do what men want and this includes serving their sexual needs
30	A man can cheat and still love his wife
31	A woman is responsible for preventing herself from being raped
32	Some children (14 to 16 years) behave in a seductive manner towards men
33	Sexual contact between a child (14 to 16 years) and a man causes the child no emotional problems
34	Sometimes children don't say no to sexual contact with a man because they are curious about sex or enjoy it
35	Women who go out often are mainly looking for sex
36	If a child (14 to 16 years) flirts with a man, it means the child wants to have sexual contact with him

Note . The presented items are translated with the utmost care, however, they do represent sample items translated to English. Reverse-scored items are denoted with an (R).

Table 1 Continued

Initial Item Pool of the Implicit Theories of Sexual Offense Questionnaire (ITSOQ)

nr.	Items
37	Most likely, children don't tell others about sexual activities with an adult man, because they liked it or weren't bothered by it
38	Most men buy erotic magazines for the interesting articles in it
39	Children (11 years or younger) can enjoy having sexual contact with a man
40	A man is entitled to have sexual contact with his (step)children, if his wife doesn't like sex
41	Nobody can be really trusted
42	The world is full of danger
43	Most men have sexual desires
44	It's a dog-eat-dog world out there
45	Most men fantasize about sex every now and then
46	In general, women want sex, regardless of how they can get it
47	It is OK to have sexual contact with a child (11 years or younger) as long as the child is not forced into it
48	Children (11 years or younger) don't have the mental capacity to decide whether to have sex with a man (R)
49	Women often flirt with men just to tease or fool them
50	A man can reliably estimate how much sexual contact between him and a child will hurt the child later on (physical and/or emotional)
51	People should mostly look after themselves
52	Any woman can prevent herself from being raped if she really wants to
53	Having sexual contact with a child is a good way to teach them about sexuality
54	Women are often partly to blame for the rape taking place
55	If a woman gets raped it will cause her harm (Physical and/or emotional) (R)
56	Children (11 years or younger) who have sexual contact with men, do not experience damage (physical and/or emotional) because of it
57	Children who are not loved by their parents are actually helped by a man who has sexual contact with them
58	Children cannot be trusted (R)
59	Women are entitled to the same freedom that is given to men (R)
60	People have no control over what happens to them in life
61	It is better to have sexual contact with your own child, than to cheat on your partner
62	Men who are forcing lonely women to have sex with them, are actually doing them a favor
63	If a woman says "no" she is just playing hard to get, but she really means "yes"
64	Sometimes a child (14 to 16 years) instigates the sexual activities with a man
65	Any woman can get raped (R)
66	Often it appears a woman cares about a man, but in reality she is only out to use that man
67	Rape serves as a way to put women in their 'place'
68	Most people are willing to help someone else in need (R)
69	Only women who have suffered physical harm during the rape have the right to file a report
70	There is no real manipulation or threat used in a lot of sexual activities by men on children
71	Normally, women can be trusted (R)
72	Some men have had sexual fantasies about someone other than their partner while in a relationship

Note . The presented items are translated with the utmost care, however, they do represent sample items translated to English. Reverse-scored items are denoted with an (R).

Table 1 Continued

Initial Item Pool of the Implicit Theories of Sexual Offense Questionnaire (ITSOQ)

nr.	Items
73	It is safer not to trust women
74	A man who just touches a child's body all over without touching the genitals of the child is not really being sexual with the child
75	Children are more reliable and more trusting than adults
76	The degree to which a woman resists should be the major factor in determining whether it is rape
77	For many men, sexual contact with children is a result of stress and the sexual activities help to relieve that stress
78	If a child (11 years or younger) stares at a man's genitals, it means the child enjoys what it sees
79	When a man spends a lot of money on a woman during a date, the woman should at least give the man something in return sexually
80	It is okay that some men involve children in sexual activities because they think the child would enjoy this
81	A woman will often pretend that she doesn't want to have sex because she does not want to be seen as easy, but she's really hoping a man will force her to have sex
82	If a child (11 years or younger) flirts with a man, it means the child wants to have sexual contact with him
83	In many cases we might just as well decide what to do by flipping a coin
84	If a woman acts like she is too good for a man, most men probably think about raping her to put her in her place
85	Women should oblige men's sexual needs
86	Only fondling the child (11 years or younger) will not cause any harm (physical and/or emotional) to the child
87	If a man does not use force to have sex with a child, it will not harm the child (physical and/or emotional)
88	In many cases men do not plan sexual activities with children, it just happens
89	Because men have high sexual needs they cannot always control their sexual urges
90	Some children (11 years or younger) behave in a seductive manner towards men
91	When it really comes down to it, many women are not trustworthy or deceitful
92	Most women have a subconscious wish to be raped
93	Children (14 to 16 years) can enjoy having sexual contact with men
94	Women who say "no" to sexual advances often mean "yes"
95	A child (11 years or younger) never fully gets over being sexually abused (R)
96	Sexual contact between a child (11 years or younger) and a man causes the child no emotional problems
97	Men rape women if they are rejected by them
98	A child (11 years or younger) that walks around naked is not instigating sexual advances towards a man (R)
99	Most men masturbate
100	Women often falsely accuse men of rape to protect their reputation
101	Many men have sex with children because they had sex with an adult man when they were a child
102	Most women manipulate to get what they want
103	For most men having sexual activities with a child is a deliberate choice (R)
104	A woman cannot expect to have the same rights as a man
105	When a man pays for dinner while on a date with a woman, he can assume that he will have sex with her later that evening
106	People can pretty much determine what will happen in their lives (R)
107	When women act like a whore, it justifies rape

Note . The presented items are translated with the utmost care, however, they do represent sample items translated to English. Reverse-scored items are denoted with an (R).

Table 1 Continued

Initial Item Pool of the Implicit Theories of Sexual Offense Questionnaire (ITSOQ)

nr.	Items
108	Women should be less concerned about their rights and pay more attention to becoming a good wife and mother
109	Any healthy woman can successfully resist during rape, if she really wants to
110	Sexual contact between a man and a child can be seen as sexual education for the child
111	A child (11 years or younger) can make its own decisions as to whether or not to have sexual contact with a man
112	Some children (14 to 16 years) are eager and willing to have sexual contact with an adult
113	Women who get raped while hitchhiking get what they deserve
114	The man is the boss in the family
115	A woman can enjoy sex even when it is forced upon her
116	Fondling a child (11 years or younger) without penetration can still cause harm (physical and/or emotional) (R)
117	A raped woman is never responsible for the rape (R)
118	Children are often able to understand emotions better than adults can
119	Children want to please adults spontaneously and without ulterior motives
120	A child (11 years or younger) that does not wear underwear and sits in a way that is revealing, gives off the impression that it wants to have sexual contact
121	Sexual contact between an adult man and a child (14 to 16 years) isn't harmful for the child (physical and/or emotional)
122	Some children (11 years or younger) are mature enough to enjoy sexual activities with men
123	Many women who got raped already had a bad reputation before that
124	Most women do not lie to get ahead in life (R)
125	Sometimes a child (11 years or younger) initiates sexual contact with a man
126	Men who force women into sexual activities often do this to get back at someone
127	Children (11 years or younger) who have been involved in sexual activities with an adult man, will eventually get over it and get on with their lives
128	Life is largely determined by random events
129	Women are usually nice until the moment they have seduced a man, then they show their true nature
130	Sexual contact with a child is not harmful for the child (physical and/or emotional) as long as the man is gentle
131	Only fondling the child (14 to 16 years) will not cause any harm (physical and/or emotional) to the child

Note . The presented items are translated with the utmost care, however, they do represent sample items translated to English. Reverse-scored items are denoted with an (R).

Table 2

Means, Standard Deviations and Ranges of the Complete Initial Item Pool

	General Population						Pedophilia-supportive Forum Users						Men Who Sexually Offended						Men Who Violently Offended					
Initial Item Pool nr.	*n*	*M*	*SD*	range	skew-ness	kurto-sis	*n*	*M*	*SD*	range	skew-ness	kurto-sis	*n*	*M*	*SD*	range	skew-ness	kurto-sis	*n*	*M*	*SD*	range	skew-ness	kurto-sis
1	424	1.25	0.66	4	3.13	10.50	20	1.45	1.05	4	2.20	4.04	28	1.32	0.86	4	3.04	9.36	21	1.29	0.90	4	3.34	10.70
2	424	1.38	0.82	4	2.41	5.39	20	2.45	1.43	4	0.45	-1.35	28	1.86	1.41	4	1.17	-0.34	21	1.33	0.80	3	2.23	3.99
3	425	1.03	0.27	4	11.90	152.00	20	1.05	0.22	1	3.82	13.30	28	1.00	0.00	0	-	-	21	1.00	0.00	0	-	-
4	424	1.23	0.65	4	3.31	11.60	20	1.05	0.22	1	3.82	13.30	28	1.21	0.83	4	3.69	13.00	21	1.19	0.87	4	3.95	14.30
5	424	1.16	0.71	4	4.76	21.90	20	1.40	1.10	4	2.41	4.44	28	1.00	0.00	0	-	-	21	1.10	0.44	2	3.95	14.30
6	424	2.59	1.21	4	0.14	-1.06	20	2.25	1.41	4	0.65	-1.02	26	2.54	1.24	4	0.28	-0.93	21	3.05	1.36	4	-0.20	-1.29
7	424	1.75	1.11	4	1.61	1.73	20	2.25	1.37	4	0.73	-0.83	28	2.07	1.54	4	1.06	-0.50	21	2.05	1.56	4	1.12	-0.46
8	424	2.07	1.06	4	0.53	-0.89	20	2.35	1.23	4	0.82	-0.27	28	1.82	1.06	4	1.07	0.52	21	1.95	1.32	4	1.07	-0.06
9	425	4.77	0.79	4	-3.97	15.40	20	4.85	0.49	2	-2.94	7.68	28	4.50	1.00	4	-1.93	3.13	21	4.29	1.42	4	-1.68	1.15
10	425	1.61	0.97	4	1.61	1.83	20	2.05	1.39	4	1.02	-0.44	28	1.54	1.20	4	2.02	2.68	21	1.48	1.08	4	2.00	3.04
11	425	1.37	0.81	4	2.34	5.05	20	2.00	1.38	4	0.92	-0.83	27	1.41	1.05	4	2.27	3.93	21	1.43	1.03	4	2.29	4.48
12	424	2.32	1.04	4	0.37	-0.65	20	2.65	1.23	4	0.32	-0.82	28	2.71	1.38	4	0.34	-1.04	21	2.76	1.18	4	-0.08	-1.11
13	423	2.18	1.20	4	0.50	-1.14	20	2.95	1.15	4	0.29	-1.19	28	1.93	1.33	4	1.03	-0.34	21	1.81	1.21	4	1.15	0.13
14	424	2.85	1.00	4	0.00	-0.56	20	2.60	0.88	3	-0.51	-0.68	27	2.74	1.26	4	0.14	-0.90	21	2.33	1.35	4	0.57	-0.98
15	424	1.71	1.01	4	1.32	0.79	20	1.40	0.75	3	2.07	4.23	28	2.07	1.44	4	0.89	-0.72	21	1.90	1.30	4	1.08	-0.35
16	424	2.55	1.18	4	0.08	-1.23	20	1.70	0.92	3	1.34	1.02	28	1.86	1.11	4	1.05	0.19	21	2.71	1.35	4	0.27	-1.10
17	425	1.49	0.90	4	1.82	2.41	20	2.40	1.64	4	0.48	-1.59	27	1.56	0.97	4	1.89	3.41	21	1.62	0.87	2	0.76	-1.28
18	424	2.33	1.21	4	0.57	-0.74	20	2.55	1.57	4	0.42	-1.40	28	2.39	1.34	4	0.53	-0.90	21	2.57	1.47	4	0.36	-1.36
19	423	1.95	1.02	4	0.78	-0.53	20	2.35	1.42	4	0.65	-1.16	28	1.93	1.36	4	1.06	-0.38	21	1.71	1.23	4	1.75	1.97
20	424	1.25	0.62	4	2.89	9.14	20	1.25	0.64	2	2.09	2.75	28	1.25	0.59	2	2.07	3.02	21	1.81	1.29	4	1.40	0.77
21	425	1.74	1.07	4	1.26	0.47	20	2.85	1.50	4	0.15	-1.49	28	2.46	1.64	4	0.42	-1.56	21	1.57	1.03	3	1.39	0.39
22	424	2.68	1.13	4	0.09	-0.97	20	2.90	1.37	4	0.17	-1.46	28	2.89	1.40	4	-0.05	-1.31	21	3.19	1.36	4	-0.10	-1.21
23	425	4.58	1.00	4	-2.63	6.05	20	4.45	1.00	4	-2.13	4.41	28	4.11	1.31	4	-1.32	0.54	21	4.38	1.02	4	-1.83	3.07
24	424	1.93	1.03	4	0.58	-1.01	20	2.90	1.21	4	-0.16	-0.76	27	2.07	1.33	4	0.82	-0.65	21	1.62	0.97	3	1.07	-0.41
25	425	1.08	0.42	4	7.13	57.60	20	1.50	1.10	4	2.14	3.49	28	1.39	1.03	4	2.33	4.26	21	1.38	1.02	4	2.44	5.06
26	425	1.11	0.39	3	3.86	16.20	20	1.00	0.00	0	-	-	28	1.18	0.67	3	3.37	10.20	21	1.29	0.72	3	2.67	6.93
27	424	2.21	1.25	4	0.50	-1.01	20	3.55	1.39	4	-0.65	-0.89	27	2.00	1.39	4	0.92	-0.70	21	2.71	1.62	4	0.31	-1.58
28	425	1.12	0.45	4	4.65	25.90	20	1.10	0.45	2	3.82	13.30	27	1.15	0.53	2	3.07	7.74	21	1.43	0.81	2	1.29	-0.24
29	423	1.02	0.21	3	11.70	143.00	20	1.25	0.79	3	2.66	5.71	28	1.29	1.05	4	3.15	8.23	21	1.00	0.00	0	-	-
30	424	3.26	1.40	4	-0.38	-1.18	20	2.85	1.42	4	0.04	-1.41	28	3.07	1.49	4	-0.31	-1.38	21	3.52	1.66	4	-0.57	-1.43
31	423	1.60	0.93	4	1.64	2.08	20	1.10	0.45	2	3.82	13.30	27	1.70	1.20	4	1.32	0.35	21	1.43	0.98	4	2.48	5.82
32	424	2.62	1.30	4	0.05	-1.41	20	3.00	1.56	4	-0.08	-1.69	28	2.36	1.34	4	0.43	-1.17	21	2.90	1.55	4	-0.16	-1.66
33	425	1.69	1.01	4	1.49	1.57	20	2.30	1.30	4	0.83	-0.55	27	1.74	1.26	4	1.25	0.02	21	1.71	1.06	3	1.04	-0.46

Note. Item descriptives related to initial item pool nr. can be found in Table 1.

Table 2 Continued

Means, Standard Deviations and Ranges of the Complete Initial Item Pool

	General Population						Pedophilia-supportive Forum Users						Men Who Sexually Offended						Men Who Violently Offended					
Initial Item Pool nr.	*n*	*M*	*SD*	range	skew-ness	kurto-sis	*n*	*M*	*SD*	range	skew-ness	kurto-sis	*n*	*M*	*SD*	range	skew-ness	kurto-sis	*n*	*M*	*SD*	range	skew-ness	kurto-sis
34	424	2.24	1.17	4	0.39	-1.08	20	3.00	1.41	4	-0.11	-1.48	27	1.74	1.29	4	1.51	0.84	21	2.33	1.43	4	0.32	-1.60
35	425	1.80	0.99	4	1.08	0.41	20	1.65	0.88	2	0.68	-1.40	27	1.78	0.97	2	0.43	-1.84	21	2.38	1.28	4	0.52	-0.76
36	424	1.48	0.72	3	1.37	1.15	20	1.90	1.33	4	1.31	0.36	27	1.52	1.05	4	1.96	2.94	21	1.67	1.15	4	1.56	1.35
37	424	1.52	0.85	4	1.49	1.21	20	1.95	1.36	4	1.17	-0.01	27	1.70	1.23	4	1.49	1.06	21	1.48	0.87	2	1.14	-0.72
38	425	4.15	1.13	4	-1.15	0.34	20	4.30	1.08	3	-1.05	-0.52	28	3.54	1.40	4	-0.27	-1.45	21	3.52	1.54	4	-0.55	-1.24
39	425	1.24	0.57	3	2.33	4.45	20	2.30	1.49	4	0.60	-1.32	28	1.57	1.17	4	1.65	1.27	21	1.29	0.72	2	1.90	1.69
40	424	1.02	0.19	2	9.38	90.80	20	1.20	0.89	4	3.82	13.30	28	1.14	0.76	4	4.74	21.20	21	1.19	0.60	2	2.56	4.81
41	425	2.48	1.34	4	0.42	-1.04	20	2.90	1.45	4	0.16	-1.43	28	3.00	1.47	4	-0.27	-1.44	21	3.10	1.48	4	-0.24	-1.45
42	425	3.45	1.28	4	-0.51	-0.82	20	3.40	1.19	4	-0.22	-1.10	28	3.86	1.35	4	-0.96	-0.38	21	3.81	1.21	4	-0.62	-0.73
43	426	4.62	0.69	4	-2.46	7.89	20	4.75	0.44	1	-1.07	-0.89	28	4.39	0.83	2	-0.79	-1.13	21	4.76	0.70	3	-3.01	8.66
44	425	3.17	1.17	4	-0.24	-0.92	20	3.05	1.32	4	-0.35	-1.21	28	3.36	1.16	4	-0.28	-0.78	21	3.81	1.21	4	-0.95	0.14
45	424	4.76	0.58	4	-3.46	15.70	20	4.80	0.41	1	-1.39	-0.07	28	4.46	0.79	2	-0.96	-0.77	21	4.86	0.48	2	-3.04	8.34
46	424	2.14	1.05	4	0.58	-0.52	20	1.80	1.15	4	1.15	0.42	28	1.79	1.07	3	0.94	-0.60	21	2.62	1.32	4	0.31	-1.22
47	426	1.04	0.30	4	9.12	95.40	20	1.45	1.10	4	2.27	3.92	28	1.29	0.85	4	3.23	10.40	21	1.43	1.21	4	2.43	4.31
48	424	1.69	1.37	4	1.80	1.58	20	1.95	1.61	4	1.24	-0.31	28	1.93	1.49	4	1.22	-0.16	21	1.38	0.97	4	2.64	6.50
49	426	2.88	1.07	4	-0.15	-0.83	20	2.50	1.10	4	0.34	-0.60	28	2.43	1.37	4	0.39	-1.08	21	3.14	0.91	4	-0.26	-0.13
50	426	1.95	1.41	4	1.12	-0.30	20	2.35	1.73	4	0.65	-1.40	28	1.89	1.40	4	1.13	-0.39	21	2.05	1.50	4	0.86	-0.99
51	426	3.20	1.20	4	-0.34	-0.90	20	3.50	1.24	4	-0.48	-0.66	28	3.50	1.43	4	-0.52	-1.13	21	3.52	1.29	4	-0.45	-0.91
52	425	1.45	0.82	4	2.09	4.12	20	1.25	0.72	3	2.91	7.99	28	1.89	1.29	4	1.00	-0.56	21	2.10	1.37	4	0.72	-1.12
53	426	1.05	0.33	4	7.81	71.70	20	1.70	1.34	4	1.52	0.85	28	1.32	0.98	4	2.74	6.27	21	1.10	0.44	2	3.95	14.30
54	426	1.34	0.64	4	2.08	4.89	20	1.15	0.49	2	2.94	7.68	28	1.32	0.91	4	2.83	7.63	21	1.33	0.58	2	1.37	0.81
55	425	1.32	0.88	4	3.19	9.66	20	1.50	1.15	4	1.99	2.64	28	1.32	0.86	4	3.04	9.36	21	1.24	0.89	4	3.62	12.30
56	426	1.08	0.40	4	6.70	53.00	20	1.85	1.39	4	1.27	0.09	28	1.29	0.85	4	3.23	10.40	21	1.19	0.87	4	3.95	14.30
57	426	1.05	0.26	2	6.02	37.70	20	1.55	1.15	4	1.88	2.32	28	1.14	0.76	4	4.74	21.20	21	1.10	0.44	2	3.95	14.30
58	426	4.06	1.14	4	-0.97	-0.11	20	4.30	0.73	2	-0.47	-1.13	28	4.07	1.27	4	-1.06	-0.07	21	4.24	1.18	4	-1.31	0.61
59	426	1.17	0.67	4	4.51	20.70	20	1.20	0.62	2	2.47	4.32	28	1.32	0.91	4	2.83	7.63	21	1.10	0.30	1	2.56	4.81
60	426	2.78	1.14	4	0.11	-0.93	20	2.90	0.85	3	0.66	-0.31	28	3.00	1.41	4	-0.23	-1.34	21	3.24	1.04	4	-0.71	-0.29
61	426	1.02	0.17	2	9.05	88.10	20	1.45	0.95	3	1.56	0.79	28	1.14	0.76	4	4.74	21.20	21	1.10	0.44	2	3.95	14.30
62	426	1.05	0.29	3	7.05	58.70	20	1.15	0.49	2	2.94	7.68	28	1.00	0.00	0	-	-	21	1.33	0.86	3	2.09	2.86
63	426	1.27	0.62	3	2.42	5.32	20	1.15	0.49	2	2.94	7.68	28	1.21	0.63	2	2.41	3.93	21	1.67	1.06	3	1.12	-0.35
64	425	2.48	1.23	4	0.11	-1.34	20	2.90	1.48	4	0.16	-1.56	27	2.19	1.39	4	0.60	-1.11	21	2.76	1.37	4	-0.03	-1.40
65	426	1.78	1.15	4	1.50	1.35	20	1.70	1.08	4	1.52	1.76	28	2.25	1.65	4	0.76	-1.14	21	1.95	1.50	4	1.18	-0.28
66	425	2.00	1.04	4	0.62	-0.78	20	2.15	1.09	4	0.65	-0.09	27	2.37	1.21	4	0.17	-1.19	21	2.33	1.15	4	0.48	-0.69

Note . Item descriptives related to initial item pool nr. can be found in Table 1.

Table 2 Continued

Means, Standard Deviations and Ranges of the Complete Initial Item Pool

	General Population						Pedophilia-supportive Forum Users						Men Who Sexually Offended						Men Who Violently Offended					
Initial Item Pool nr.	*n*	*M*	*SD*	range	skew-ness	kurto-sis	*n*	*M*	*SD*	range	skew-ness	kurto-sis	*n*	*M*	*SD*	range	skew-ness	kurto-sis	*n*	*M*	*SD*	range	skew-ness	kurto-sis
67	426	1.19	0.66	4	3.68	13.40	20	1.15	0.49	2	2.94	7.68	28	1.07	0.38	2	4.74	21.20	21	1.29	0.78	3	2.47	4.99
68	426	1.92	0.93	4	1.23	1.61	20	2.15	0.88	3	0.63	-0.27	28	1.93	1.05	3	0.69	-0.91	21	1.95	1.07	3	0.79	-0.74
69	425	1.28	0.98	4	3.41	9.91	20	1.25	0.79	3	2.66	5.71	28	1.57	1.43	4	1.93	1.80	21	1.10	0.44	2	3.95	14.30
70	426	1.44	0.95	4	2.31	4.62	20	1.90	1.25	4	0.94	-0.42	27	1.78	1.45	4	1.39	0.20	21	2.10	1.58	4	0.87	-1.00
71	425	1.82	1.01	4	1.25	1.04	20	2.00	0.97	3	0.65	-0.66	27	2.37	1.45	4	0.54	-1.00	21	2.29	1.19	4	0.49	-0.80
72	426	4.52	0.77	4	-2.14	5.89	20	4.40	0.88	3	-1.24	0.51	28	4.32	0.82	2	-0.61	-1.29	21	4.52	0.60	2	-0.74	-0.60
73	424	1.74	0.98	4	1.22	0.74	20	1.90	1.02	3	0.47	-1.40	28	2.18	1.22	4	0.62	-0.49	21	1.81	1.12	4	1.36	1.05
74	426	1.29	0.63	3	2.39	5.66	20	2.15	1.35	4	0.97	-0.43	27	1.37	0.93	4	2.60	6.49	21	1.38	1.07	4	2.50	4.92
75	426	2.95	1.29	4	-0.11	-1.01	20	3.30	1.17	4	-0.37	-0.68	28	2.89	1.31	4	0.00	-1.04	21	3.14	1.31	4	0.00	-0.98
76	425	1.55	1.02	4	1.90	2.74	20	1.45	0.83	2	1.22	-0.44	28	2.00	1.52	4	1.05	-0.55	21	1.76	1.18	3	0.96	-0.86
77	426	1.46	0.82	4	1.51	1.06	20	1.85	1.09	4	1.21	0.93	27	1.81	1.27	4	1.09	-0.35	21	1.33	0.73	2	1.66	0.94
78	426	1.09	0.47	4	6.03	40.30	20	1.70	1.22	4	1.38	0.60	28	1.21	0.79	4	4.03	16.20	21	1.24	0.89	4	3.62	12.30
79	426	1.15	0.51	4	3.97	17.70	20	1.25	0.64	2	2.09	2.75	28	1.21	0.57	2	2.36	4.25	21	1.33	0.97	4	2.83	7.33
80	426	1.04	0.25	3	7.31	62.80	20	1.55	1.10	4	1.80	2.36	28	1.29	0.85	4	3.23	10.40	21	1.10	0.44	2	3.95	14.30
81	426	1.32	0.66	3	2.11	3.85	20	1.25	0.64	2	2.09	2.75	28	1.36	0.73	2	1.58	0.73	21	1.71	0.85	2	0.53	-1.45
82	426	1.10	0.40	3	4.54	22.00	20	1.50	1.00	4	2.25	4.81	28	1.21	0.79	4	4.03	16.20	21	1.05	0.22	1	3.95	14.30
83	424	1.49	0.90	4	1.85	2.63	20	1.70	1.08	4	1.52	1.76	28	1.54	1.04	4	1.74	2.24	21	1.52	0.93	3	1.36	0.40
84	426	1.19	0.54	3	3.23	10.60	20	1.20	0.62	2	2.47	4.32	28	1.21	0.63	2	2.41	3.93	21	1.52	0.98	3	1.60	1.18
85	426	1.15	0.52	4	4.52	24.40	20	1.45	0.83	2	1.22	-0.44	28	1.07	0.38	2	4.74	21.20	21	1.67	0.86	2	0.64	-1.39
86	426	1.34	0.72	4	2.59	7.43	20	2.50	1.47	4	0.47	-1.25	28	1.50	1.07	4	1.92	2.57	21	1.33	0.73	2	1.66	0.94
87	425	1.07	0.30	2	4.79	23.80	20	1.65	1.18	4	1.57	1.24	28	1.36	1.10	4	2.71	5.91	21	1.19	0.87	4	3.95	14.30
88	426	1.71	0.91	3	0.86	-0.64	20	2.20	1.11	4	0.74	-0.10	28	1.89	1.29	4	1.20	0.14	21	1.67	1.20	4	1.46	0.84
89	426	2.12	1.21	4	0.64	-0.92	20	2.10	1.21	4	0.84	-0.42	28	2.21	1.34	4	0.60	-1.00	21	2.14	1.31	4	0.63	-1.06
90	426	1.32	0.75	4	2.37	4.82	20	2.35	1.42	4	0.55	-1.20	28	1.57	1.07	4	1.75	2.13	21	1.38	0.81	3	2.01	3.21
91	425	1.68	0.92	3	1.16	0.24	20	1.75	0.91	2	0.47	-1.68	28	1.82	1.16	4	1.03	-0.09	21	2.33	1.35	4	0.68	-0.92
92	426	1.20	0.60	4	3.24	10.70	20	1.15	0.49	2	2.94	7.68	28	1.39	0.79	2	1.45	0.18	21	1.52	1.12	4	1.96	2.67
93	426	2.31	1.25	4	0.35	-1.30	20	3.10	1.37	4	0.06	-1.53	28	2.04	1.35	4	0.82	-0.73	21	2.10	1.34	4	0.67	-1.08
94	424	1.22	0.50	3	2.36	5.41	20	1.25	0.64	2	2.09	2.75	28	1.36	0.78	2	1.59	0.55	21	1.62	0.92	3	1.13	-0.01
95	426	1.93	1.23	4	1.31	0.67	20	2.95	1.19	4	0.09	-1.13	28	1.82	1.47	4	1.45	0.39	21	1.90	1.30	4	1.34	0.53
96	425	1.21	0.73	4	4.02	16.40	20	1.90	1.21	4	1.03	-0.08	28	1.46	1.14	4	2.27	3.92	21	1.48	0.98	3	1.73	1.50
97	426	1.95	1.09	4	0.72	-0.72	20	1.65	0.93	2	0.69	-1.53	28	2.07	1.18	4	0.64	-0.74	21	1.71	1.15	3	1.11	-0.49
98	424	1.55	1.26	4	2.11	2.84	20	2.05	1.61	4	0.94	-0.95	28	1.89	1.57	4	1.27	-0.18	21	2.05	1.66	4	1.00	-0.88
99	426	4.61	0.80	4	-2.54	6.87	20	4.75	0.64	2	-2.09	2.75	28	4.57	0.92	4	-2.40	5.77	21	4.24	0.89	2	-0.45	-1.64

Note . Item descriptives related to initial item pool nr. can be found in Table 1.

Table 2 Continued

Means, Standard Deviations and Ranges of the Complete Initial Item Pool

Initial Item Pool nr.	General Population						Pedophilia-supportive Forum Users						Men Who Sexually Offended						Men Who Violently Offended					
	n	*M*	*SD*	range	skew-ness	kurto-sis	*n*	*M*	*SD*	range	skew-ness	kurto-sis	*n*	*M*	*SD*	range	skew-ness	kurto-sis	*n*	*M*	*SD*	range	skew-ness	kurto-sis
100	426	1.99	1.03	4	0.66	-0.59	20	2.20	1.20	4	0.51	-0.77	27	2.00	1.33	4	0.94	-0.47	21	2.76	1.09	3	-0.43	-1.20
101	425	2.23	1.13	4	0.21	-1.26	20	1.85	1.23	4	1.08	-0.05	28	2.57	1.55	4	0.30	-1.42	21	2.14	1.39	4	0.72	-0.86
102	425	2.27	1.19	4	0.43	-1.06	20	1.85	0.93	3	0.65	-0.82	28	2.04	1.20	4	0.68	-0.77	21	2.76	1.26	4	0.43	-0.91
103	425	2.35	1.32	4	0.79	-0.50	20	2.40	1.14	4	0.44	-0.68	28	2.89	1.55	4	0.17	-1.53	21	2.14	1.65	4	0.86	-1.07
104	425	1.13	0.54	4	5.26	30.90	20	1.10	0.45	2	3.82	13.30	28	1.29	0.90	4	3.01	8.47	21	1.57	1.03	4	1.92	3.31
105	426	1.12	0.48	4	4.91	28.40	20	1.10	0.45	2	3.82	13.30	28	1.18	0.55	2	2.71	5.91	21	1.24	0.54	2	2.00	3.04
106	426	2.11	1.01	4	1.13	1.06	20	2.50	1.00	4	0.45	0.01	28	1.93	1.15	4	1.25	0.93	21	2.05	0.97	4	1.15	1.59
107	426	1.13	0.42	3	3.62	15.10	20	1.30	0.80	3	2.38	4.50	28	1.32	0.91	4	2.83	7.63	21	1.14	0.48	2	3.04	8.34
108	426	1.54	0.94	4	1.70	2.05	20	1.25	0.64	2	2.09	2.75	28	1.64	1.13	4	1.44	0.92	21	2.24	1.58	4	0.86	-0.96
109	426	1.46	0.90	4	2.14	3.99	20	1.25	0.55	2	1.91	2.67	28	1.75	1.38	4	1.34	0.09	21	1.76	1.22	4	1.38	0.58
110	426	1.08	0.40	3	5.21	28.40	20	1.60	1.27	4	1.90	2.20	28	1.29	0.94	4	3.08	8.34	21	1.19	0.60	2	2.56	4.81
111	426	1.11	0.44	4	5.40	35.20	20	1.50	1.15	4	1.99	2.64	28	1.36	0.95	4	2.76	6.85	21	1.57	1.25	4	1.68	1.20
112	426	2.63	1.33	4	0.06	-1.39	20	3.15	1.35	4	-0.26	-1.34	28	1.93	1.36	4	1.06	-0.38	21	2.57	1.57	4	0.25	-1.61
113	426	1.07	0.32	4	6.81	61.20	20	1.10	0.45	2	3.82	13.30	28	1.11	0.42	2	3.69	13.00	21	1.10	0.44	2	3.95	14.30
114	424	1.63	1.05	4	1.49	0.96	20	1.40	0.82	2	1.39	-0.07	28	1.50	1.17	4	2.07	3.02	21	2.10	1.37	4	0.72	-1.12
115	426	1.77	1.06	4	1.11	0.00	20	1.60	0.88	3	1.24	0.51	28	1.43	0.92	4	2.40	5.77	21	2.10	1.37	4	0.72	-1.12
116	426	1.70	1.26	4	1.81	1.95	20	2.05	1.39	4	0.80	-1.05	28	2.32	1.76	4	0.77	-1.32	21	2.24	1.81	4	0.81	-1.33
117	426	1.99	1.34	4	1.12	-0.14	20	2.25	1.71	4	0.82	-1.18	27	2.15	1.70	4	0.90	-1.07	21	1.86	1.31	4	1.38	0.58
118	422	1.74	1.02	4	1.16	0.43	20	2.50	1.47	4	0.47	-1.25	28	2.14	1.35	4	0.79	-0.56	21	1.86	1.28	4	1.08	-0.27
119	424	3.51	1.19	4	-0.70	-0.25	20	3.65	1.35	4	-0.61	-0.92	28	3.25	1.46	4	-0.28	-1.17	21	3.43	1.69	4	-0.42	-1.61
120	426	1.08	0.36	3	5.44	32.30	20	1.65	1.23	4	1.62	1.20	28	1.18	0.77	4	4.36	18.40	21	1.05	0.22	1	3.95	14.30
121	426	1.55	0.87	4	1.60	2.08	20	2.55	1.23	4	0.53	-0.74	28	1.57	1.23	4	1.86	2.02	21	1.48	0.81	2	1.13	-0.56
122	426	1.08	0.32	2	4.38	19.90	20	1.85	1.42	4	1.29	-0.01	28	1.32	0.95	4	2.91	7.54	21	1.10	0.44	2	3.95	14.30
123	426	1.17	0.54	3	3.09	8.59	20	1.15	0.49	2	2.94	7.68	28	1.43	0.96	4	2.26	4.67	21	1.14	0.36	1	1.90	1.69
124	425	2.75	1.24	4	0.13	-0.94	20	3.10	1.37	4	-0.17	-1.12	28	2.82	1.42	4	0.15	-1.25	21	3.00	1.00	4	-0.29	-0.33
125	426	1.24	0.60	3	2.47	5.20	20	2.00	1.41	4	0.85	-1.03	28	1.36	1.06	4	2.51	4.71	21	1.29	0.64	2	1.86	1.97
126	425	1.89	0.94	3	0.49	-1.12	20	2.00	1.03	3	0.28	-1.56	28	2.04	1.14	3	0.37	-1.54	21	2.10	1.41	4	0.86	-0.76
127	426	1.58	0.89	4	1.40	1.01	20	2.30	1.13	4	0.69	-0.38	28	1.50	1.07	4	1.92	2.57	21	1.52	0.75	2	0.94	-0.67
128	424	2.97	1.10	4	-0.22	-0.87	20	3.15	1.04	4	-0.55	-0.37	28	2.86	1.21	4	-0.22	-0.91	21	3.43	1.16	4	-0.47	-0.44
129	424	1.74	1.00	4	1.23	0.63	20	1.60	0.88	2	0.80	-1.27	28	1.89	1.17	4	0.88	-0.39	21	2.19	1.29	4	0.60	-1.03
130	424	1.07	0.36	4	6.56	52.00	20	1.75	1.33	4	1.58	1.01	28	1.32	0.86	4	3.04	9.36	21	1.14	0.48	2	3.04	8.34
131	423	1.47	0.79	4	1.76	2.60	20	2.35	1.35	4	0.61	-0.92	28	1.61	1.17	4	1.58	1.11	21	1.52	0.87	2	1.01	-0.96

Note . Item descriptives related to initial item pool nr. can be found in Table 1.

Table .3

Difference Between the Items with the Different Age Categories (≤ 11 years versus 14-16 years) for the General Population Sample (N = 426)

Item Description	14-16 years			≤ 11 years			Wilcoxon Signed-ranks test
	M (*SD*)	Range	*Mdn*	*M* (*SD*)	Range	*Mdn*	
Some children (age) are eager or willing to have sexual contact with an adult	2.63 (1.33)	4	3	1.37 (0.81)	4	1	**Z = -13.34, p = <.001, r = .65**
Some children (age) are mature enough to enjoy sexual activities with men	2.18 (1.2)	4	2	1.08 (0.32)	2	1	**Z = - 13.46, p = <.001, r = .65**
A child (age) can make its own decisions as to whether or not to have sexual contact with a man	1.95 (1.02)	4	2	1.11 (0.44)	4	1	**Z = -12.81, p = <.001, r = .62**
Some children (age) behave in a seductive manner towards men	2.62 (1.3)	4	3	1.32 (0.75)	4	1	**Z = -13.88, p = <.001, r = .67**
If a child (age) flirts with a man, it means the child wants to have sexual contact with him	1.48 (0.72)	3	1	1.10 (0.40)	3	1	**Z = -9.49, p = <.001, r = .46**
Children (age) can enjoy having sexual contact with a man	2.31 (1.25)	4	2	1.24 (0.57)	3	1	**Z = - 13.12, p = <.001, r = .64**
Sometimes a child (age) instigates the sexual activities with a man	2.48 (1.23)	4	3	1.24 (0.60)	3	1	**Z = -13.94, p = <.001, r = .68**
Children (age) who have been involved in sexual activities with an adult man, will eventually get over it and get on with their lives	2.07 (1.06)	4	2	1.58 (0.89)	4	1	**Z = -8.83, p = <.001, r = .43**
It is OK to have sexual contact with a child (age) as long as the child is not forced into it	1.61 (0.97)	4	1	1.04 (0.3)	4	1	**Z = -10.57, p = <.001, r = .51**
Sexual contact between an adult and child (age) isn't harmful for the child (physical and/or emotional)	1.55 (0.87)	4	1	1.08 (0.42)	4	1	**Z = -9.49, p = <.001, r = .46**
Sexual contact between a child (age) and a man causes the child no emotional problems	1.69 (1.01)	4	1	1.21 (0.73)	4	1	**Z = -8.66, p = <.001, r = .41**
Only fondling the child (age) will not cause any harm (physical and/or emotional) to the child	1.34 (0.72)	4	1	1.47 (0.79)	4	1	**Z = -3.52, p = <.001, r = .17**

Note . Significant values (p ≤.05) are in **bold**.

Table 4

Difference Between the Items with the Different Age Categories (≤ 11 years versus 14-16 years) for the Pedophilia-supportive Forum Users (N = 20)

Item Description	14-16 years			≤ 11 years			Wilcoxon Signed-ranks test
	M (*SD*)	Range	*Mdn*	*M* (*SD*)	range	*Mdn*	
Some children (age) are eager or willing to have sexual contact with an adult	3.15 (1.35)	4	3.5	2.00 (1.38)	4	1	**Z = -3.19, p = <.05 r = .15**
Some children (age) are mature enough to enjoy sexual activities with men	2.95 (1.15)	4	3	1.08 (0.32)	2	1	**Z = -3.50, p = <.05, r = .17**
A child (age) can make its own decisions as to whether or not to have sexual contact with a man	2.35 (1.42)	4	2	1.11 (0.44)	4	1	**Z = -2.82, p = <.05 r = .14**
Some children (age) behave in a seductive manner towards men	3.00 (1.56)	4	3.5	1.32 (0.75)	4	2	**Z = -2.30 p = <.05, r = .11**
If a child (age) flirts with a man, it means the child wants to have sexual contact with him	1.90 (1.33)	4	1	1.10 (0.40)	3	1	Z = -1.91, p = 056, r = .09
Children (age) can enjoy having sexual contact with a man	3.10 (1.37)	4	3	1.24 (0.57)	3	2	**Z = -2.91, p = <.05, r = .14**
Sometimes a child (age) instigates the sexual activities with a man	2.90 (1.48)	4	2.5	1.24 (0.60)	3	1	**Z = -2.16, p = <.05, r = .10**
Children (age) who have been involved in sexual activities with an adult man, will eventually get over it and get on with their lives	2.35 (1.23)	4	2	1.58 (0.89)	4	2	Z =-0.22, p = 824, r = .01
It is OK to have sexual contact with a child (age) as long as the child is not forced into it	2.05 (1.39)	4	1.5	1.04 (0.30)	4	1	**Z = -2.74, p = <.05, r = .13**
Sexual contact between an adult and child (age) isn't harmful for the child (physical and/or emotional)	2.55 (1.23)	4	2	1.08 (0.42)	4	1	**Z = -3.21 p = <.05, r = .16**
Sexual contact between a child (age) and a man causes the child no emotional problems	2.30 (1.30)	4	2	1.21 (0.73)	4	1	Z = -1.29, p = 195, r = .06
Only fondling the child (age) will not cause any harm (physical and/or emotional) to the child	2.50 (1.47)	4	2	1.47 (0.79)	4	2	Z = - 0.27, p = 784, r = .01

Note. Significant values (p ≤.05) are in **bold**.

Table 5

Difference Between the Items with the Different Age Categories (≤ 11 years versus 14-16 years) for the Men Who Sexually Offended Sample (N = 28)

Item Description	14-16 years			≤ 11 years			Wilcoxon Signed-ranks test
	M (*SD*)	Range	*Mdn*	*M* (*SD*)	Range	*Mdn*	
Some children (age) are eager or willing to have sexual contact with an adult	1.93 (1.36)	4	1	1.41 (1.05)	4	1	**Z = -2.48 p = <.05, r = .12**
Some children (age) are mature enough to enjoy sexual activities with men	1.93 (1.33)	4	1	1.32 (0.95)	4	1	**Z = - 2.64, p = <.05, r = .13**
A child (age) can make its own decisions as to whether or not to have sexual contact with a man	1.93 (1.36)	4	1	1.36 (0.95)	4	1	**Z = -2.64, p = <.05, r = .13**
Some children (age) behave in a seductive manner towards men	2.36 (1.34)	4	2	1.57 (1.07)	4	1	**Z = -3.09, p = <.05 r = .15**
If a child (age) flirts with a man, it means the child wants to have sexual contact with him	1.52 (1.05)	4	1	1.21 (0.79)	4	1	Z = -1.92, p = 054, r = .09
Children (age) can enjoy having sexual contact with a man	2.04 (1.35)	4	1	1.57 (1.17)	4	1	**Z = -2.05, p = <.05 r = .10**
Sometimes a child (age) instigates the sexual activities with a man	2.19 (1.39)	4	1	1.36 (1.06)	4	1	**Z = -2.98, p = <.05, r = .14**
Children (age) who have been involved in sexual activities with an adult man, will eventually get over it and get on with their lives	1.82 (1.06)	4	1	1.50 (1.07)	4	1	**Z = -2.25, p = <.05, r = .11**
It is OK to have sexual contact with a child (age) as long as the child is not forced into it	1.54 (1.20)	4	1	1.29 (0.85)	4	1	Z = -1.93, p = 053, r = .09
Sexual contact between an adult and child (age) isn't harmful for the child (physical and/or emotional)	1.57 (1.23)	4	1	1.39 (1.03)	4	1	Z = -0.63, p = .523, r = .03
Sexual contact between a child (age) and a man causes the child no emotional problems	1.74 (1.26)	4	1	1.46 (1.14)	4	1	Z = -0.85, p = .394, r = .04
Only fondling the child (age) will not cause any harm (physical and/or emotional) to the child	1.50 (1.07)	4	1	1.61 (1.17)	4	1	Z = -0.51, p = .608,, r = .02

Note. Significant values (p ≤.05) are in **bold**.

Table 6

Difference Between the Items with the Different Age Categories (≤ 11 years versus 14-16 years) for the Men Who Violently Offended Sample (N = 21)

	14-16 years			≤ 11 years			
Item Description	*M* (*SD*)	Range	*Mdn*	*M* (*SD*)	Range	*Mdn*	Wilcoxon Signed-ranks test
Some children (age) are eager or willing to have sexual contact with an adult	2.57 (1.57)	4	3	1.43 (1.03)	4	1	**Z = -2.80, p = <.05, r = .14**
Some children (age) are mature enough to enjoy sexual activities with men	1.81 (1.21)	4	1	1.10 (0.44)	2	1	**Z = -2.52, p = <.05, r = .12**
A child (age) can make its own decisions as to whether or not to have sexual contact with a man	1.71 (1.23)	4	1	1.57 (1.25)	4	1	*Z* = -0.70, *p* = 482, *r* = .03
Some children (age) behave in a seductive manner towards men	2.90 (1.55)	4	3	1.38 (0.81)	3	1	**Z = -3.22, p = <.05, r = .16**
If a child (age) flirts with a man, it means the child wants to have sexual contact with him	1.67 (1.15)	4	1	1.05 (0.22)	1	1	**Z = -2.32, p = <.05, r = .11**
Children (age) can enjoy having sexual contact with a man	2.10 (1.34)	4	1	1.29 (0.72)	2	1	**Z = -2.64, p = <.05, r = .13**
Sometimes a child (age) instigates the sexual activities with a man	2.76 (1.37)	4	3	1.29 (0.64)	2	1	**Z = -3.39, p = <.001, r = .16**
Children (age) who have been involved in sexual activities with an adult man, will eventually get over it and get on with their lives	1.95 (1.32)	4	1	1.52 (0.75)	2	1	**Z = -1.38, p = 168, r = .07**
It is OK to have sexual contact with a child (age) as long as the child is not forced into it	1.48 (1.08)	4	1	1.43 (1.21)	4	1	**Z = -0.18, p = 854, r = .01**
Sexual contact between an adult and child (age) isn't harmful for the child (physical and/or emotional)	1.48 (0.81)	2	1	1.38 (1.02)	4	1	**Z = -0.54, p = .586, r = .03**
Sexual contact between a child (age) and a man causes the child no emotional problems	1.71 (1.06)	3	1	1.48 (0.98)	3	1	**Z = -0.71, p = 479, r = .03**
Only fondling the child (age) will not cause any harm (physical and/or emotional) to the child	1.33 (0.73)	2	1	1.52 (0.87)	2	1	**Z = -1.11, p = 265, r = .05**

Note. Significant values (*p* ≤.05) are in **bold**.

3. Principle Component Analysis

Online supplementary material 3 is complementary to the Principle Component Analysis in the result section of the full paper. The file contains the items included in the PCA, the results of the PCA for the 4-, 5- and 6-component solutions including the pattern and structure matrix.

Table 7

Items included in the Principle Component Analysis of the Implicit Theories of Sexual Offense Questionnaire (ITSOQ)

Nr.	Initial Item Pool nr.	Item Description
1	6	Women are sly and manipulating when they want to seduce a man
2	7	Fondling a child can be considered as being sexual with a child (R)
3	8	Children (14 to 16 years) who have been involved in sexual activities with an adult male, will eventually get over it and get on with their lives
4	9	Most men think about sex at least once a month
5	10	It is OK to have sexual contact with a child (14 to 16 years) as long as the child is not forced into it
6	12	Women often falsely accuse men of rape
7	13	Some children (14 to 16 years) are mature enough to enjoy sexual activities with men
8	14	Men rarely become suspicious of women who are friendlier than they expect
9	16	If a woman goes home with a man on the first date, she probably wants to have sex with him
10	17	It is society's reaction, rather than the sexual activities itself, which causes the distress a child feels
11	18	Many people are out to hurt each other
12	19	A child (14 to 16 years) can make its own decisions as to whether or not to have sexual contact with a man
13	21	Some men are not "true" child molesters - they have just lost control and made a mistake
14	22	Most things in life just happen to you
15	23	Most men have read an erotic magazine or visited a porn website at least once in their life
16	24	Some men turn to sexual contact with children because they are deprived of sex with their adult partner
17	27	Children are more accepting and loving than adults are
18	30	A man can cheat and still love his wife
19	32	Some children (14 to 16 years) behave in a seductive manner towards men
20	33	Sexual contact between a child (14 to 16 years) and a man causes the child no emotional problems
21	34	Sometimes children don't say no to sexual contact with a man because they are curious about sex or enjoy it
22	38	Most men buy erotic magazines for the interesting articles in it
23	41	Nobody can be really trusted
24	42	The world is full of danger
25	44	It's a dog-eat-dog world out there
26	46	In general, women want sex, regardless of how they can get it
27	49	Women often flirt with men just to tease or fool them
28	50	A man can reliably estimate how much sexual contact between him and a child will hurt the child later on (physical and/or emotional)
29	51	People should mostly look after themselves
30	60	People have no control over what happens to them in life
31	64	Sometimes a child (14 to 16 years) instigates the sexual activities with a man
32	65	Any woman can get raped (R)
33	66	Often it appears a woman cares about a man, but in reality she is only out to use that man

Note . The presented items are translated with the utmost care, however, they do represent sample items translated to English. Reverse-scored items are denoted with an (R).

Table 7 Continued

Items included in the Principle Component Analysis of the Implicit Theories of Sexual Offense Questionnaire (ITSOQ)

Nr.	Initial Item Pool nr.	Item Description
34	68	Most people are willing to help someone else in need (R)
35	71	Normally, women can be trusted (R)
36	72	Some men have had sexual fantasies about someone other than their partner while in a relationship
37	73	It is safer not to trust women
38	75	Children are more reliable and more trusting than adults
39	88	In many cases men do not plan sexual activities with children, it just happens
40	89	Because men have high sexual needs they cannot always control their sexual urges
41	95	A child (11 years or younger) never fully gets over being sexually abused (R)
42	98	A child (11 years or younger) that walks around naked is not instigating sexual advances towards a man (R)
43	99	Most men masturbate
44	100	Women often falsely accuse men of rape to protect their reputation
45	101	Many men have sex with children because they had sex with an adult man when they were a child
46	102	Most women manipulate to get what they want
47	103	For most men having sexual activities with a child is a deliberate choice (R)
48	106	People can pretty much determine what will happen in their lives (R)
49	112	Some children (14 to 16 years) are eager and willing to have sexual contact with an adult
50	116	Fondling a child (11 years or younger) without penetration can still cause harm (physical and/or emotional) (R)
51	117	A raped woman is never responsible for the rape (R)
52	118	Children are often able to understand emotions better than adults can
53	119	Children want to please adults spontaneously and without ulterior motives
54	121	Sexual contact between an adult man and a child (14 to 16 years) isn't harmful for the child (physical and/or emotional)
55	124	Most women do not lie to get ahead in life (R)
56	127	Children (11 years or younger) who have been involved in sexual activities with an adult man, will eventually get over it and get on with their
57	128	Life is largely determined by random events
58	131	Only fondling the child (14 to 16 years) will not cause any harm (physical and/or emotional) to the child

Note . The presented items are translated with the utmost care, however, they do represent sample items translated to English. Reverse-scored items are denoted with an (R).

Determining the number of components

The number of components was determined using the scree test (Cattell, 1966), combined with parallel analysis (Horn, 1965; Turner, 1998). The scree test involves examining the graph of the eigenvalues for a natural bend, indicative of a break point where the curve flattens out. With regard to parallel analysis, if the eigenvalue of communality exceeds the average eigenvalue of the corresponding factor in parallel random data, the component is retained (Hayton et al., 2004). Besides the results of the scree plot and the parallel analysis, parsimony, theoretical convergence and reliability of the scales were also considered as criteria.

Figure 1. Scree plot and parallel analysis

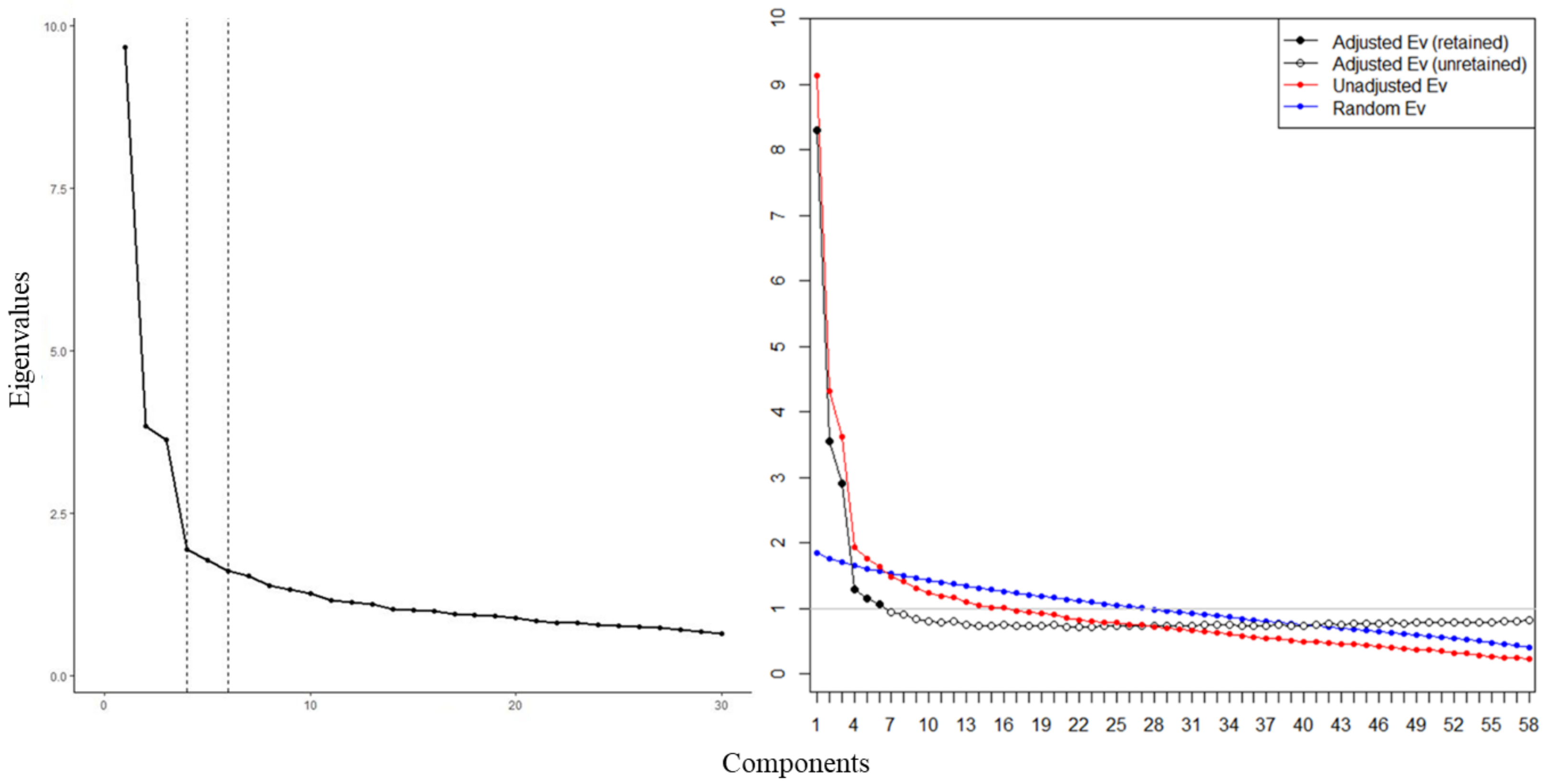

Table 8

Results of the Principal Component Analyses of the Four Component Solution in the General Population Sample (N = 427)

Item Nr.	Component Loadings				h^2	Δ Primary-Secondary Loading	Selected Item based on Component Loading Rules[1]
	1	2	3	4			
1	0.04	**0.68**	0.11	-0.01	**0.49**	0.57	2
2	0.29	0.01	-0.47	-0.01	0.29	0.18	
3	**0.63**	0.09	0.04	-0.12	**0.46**	0.51	1
4	0.10	0.00	**0.54**	-0.06	0.31	0.44	3
5	**0.70**	-0.05	-0.10	0.04	**0.47**	0.60	1
6	0.07	**0.68**	0.02	-0.07	**0.48**	0.61	2
7	**0.81**	-0.04	0.04	0.03	**0.64**	0.77	1
8	0.13	0.01	0.15	0.01	0.04		
9	0.24	0.32	0.22	-0.23	0.28		
10	0.31	0.30	-0.22	-0.06	0.29		
11	0.01	0.36	0.01	0.33	0.28		
12	**0.69**	0.08	-0.01	0.12	**0.55**	0.57	1
13	**0.42**	0.02	-0.11	0.02	0.19	0.31	1
14	0.30	-0.03	-0.01	**0.54**	0.38	0.24	4
15	0.13	0.01	**0.53**	0.05	0.32	0.40	3
16	0.30	0.10	-0.02	0.08	0.13		
17	0.18	0.24	0.08	0.10	0.15		
18	0.34	0.09	0.31	-0.12	0.25		
19	**0.63**	0.08	0.14	0.03	**0.47**	0.49	1
20	**0.70**	-0.01	-0.08	0.03	**0.49**	0.62	1
21	**0.62**	0.07	0.08	0.06	**0.44**	0.54	1
22	0.05	-0.16	0.25	-0.16	0.12		
23	-0.10	**0.44**	0.00	0.38	0.39	0.06	
24	-0.08	0.27	0.22	**0.50**	**0.43**	0.23	4
25	-0.06	**0.47**	0.14	0.27	0.35	0.20	2
26	0.04	**0.49**	-0.02	0.11	0.29	0.38	2
27	0.01	**0.49**	0.13	0.19	0.34	0.30	2
28	0.07	0.21	-0.02	-0.19	0.08		
29	-0.02	**0.43**	0.07	0.21	0.26	0.22	2
30	0.05	0.04	-0.10	**0.69**	**0.49**	0.59	4
31	**0.70**	0.04	0.14	0.05	**0.55**	0.56	1
32	0.05	0.06	-0.31	-0.13	0.13		
33	0.04	**0.60**	-0.04	0.12	**0.43**	0.48	2
34	0.05	0.35	-0.34	0.11	0.27		
35	-0.05	**0.68**	-0.27	-0.04	**0.52**	0.41	2
36	0.19	0.02	**0.60**	-0.07	**0.41**	0.41	3
37	-0.05	**0.64**	-0.09	0.08	**0.42**	0.55	2
38	0.00	0.08	0.16	0.12	0.06		
39	0.27	0.10	-0.06	0.06	0.11		
40	0.22	0.37	0.11	-0.01	0.26		

Note. The extraction method was principal component analysis with an oblique rotation. [1]Selection based on component loading rules (primary loading >.40; secundary loading <.30, difference primary - secundary loading >.20). Results are based on the pattern matrix. Results from the structural matrix did not diverge based on the final model obtained. Item number corresponds with the item number in Table 7. h^2 = communalities. Δ primary - secundary loading = difference between the primary and secundary loading.

Table 8 Continued

Results of the Principal Component Analyses of the Four Component Solution in the General Population Sample (N = 427)

Item Nr.	Component Loadings				h^2	Δ Primary-Secondary Loading	Selected Item based on Component Loading Rules[1]
	1	2	3	4			
41	0.25	0.15	-0.34	-0.30	0.30		
42	0.03	0.22	-0.37	-0.15	0.21		
43	0.17	0.09	**0.56**	0.04	0.38	0.39	3
44	0.05	**0.69**	0.07	-0.07	**0.49**	0.62	2
45	0.17	0.06	0.12	0.07	0.06		
46	0.13	**0.69**	0.10	0.00	**0.57**	0.56	2
47	0.03	-0.05	-0.38	-0.04	0.15		
48	0.11	-0.16	-0.45	0.34	0.29	0.11	
49	**0.72**	0.06	0.17	-0.01	**0.60**	0.55	1
50	0.15	0.18	-0.48	-0.11	0.31	0.30	3
51	0.12	0.38	-0.29	-0.17	0.28		
52	0.12	-0.01	-0.18	0.29	0.12		
53	0.02	0.00	0.34	0.03	0.12		
54	**0.76**	-0.10	-0.05	0.03	**0.53**	0.66	1
55	-0.08	**0.53**	-0.14	-0.06	0.27	0.39	2
56	**0.47**	0.07	-0.11	-0.22	0.29	0.25	1
57	0.11	0.05	0.01	**0.60**	0.40	0.49	4
58	**0.60**	0.04	-0.24	-0.09	**0.42**	0.36	1
Coefficient Alpha	.89 (95% CI = .87-.90)	.83 (95% CI = .80-.85)	.69 (95% CI = .64-.74)	.66 (95% CI = ..60-.71)			
Proportion of variance accounted for (%)	12	10	6	5			

Note. The extraction method was principal component analyses with an oblique rotation. [1]Selection based on component loading rules (primary loading >.40; secundary loading <.30, difference primary - secundary loading >.20). Results are based on the pattern matrix. Results from the structural matrix did not diverge based on the final model obtained. Item number corresponds with the item number in Table 7. h^2 = communalities. Δ primary - secundary loading = difference between the primary and secundary loading.

Table 9

Results of the Principal Component Analyses of the Five Component Solution in the General Population Sample (N = 427)

Item Nr.	Component Loadings					h^2	Δ Primary-Secondary Loading	Selected Item based on Component Loading Rules[1]
	1	2	3	4	5			
1	0.05	**0.68**	0.14	0.01	-0.02	0.50	0.54	2
2	0.29	0.02	-0.46	-0.01	-0.01	0.30	0.17	
3	**0.63**	0.10	0.07	-0.12	0.01	0.46	0.51	1
4	0.14	0.02	**0.60**	-0.03	-0.14	0.37	0.46	3
5	**0.71**	-0.04	-0.06	0.05	-0.03	0.49	0.65	1
6	0.05	**0.67**	0.02	-0.07	0.08	0.49	0.59	2
7	**0.79**	-0.03	0.05	0.03	0.06	0.64	0.73	1
8	0.06	-0.01	0.07	-0.02	0.28	0.10		
9	0.20	0.32	0.19	-0.24	0.14	0.29		
10	0.27	0.29	-0.25	-0.08	0.17	0.31		
11	0.03	0.36	0.05	0.35	-0.07	0.30		
12	**0.71**	0.09	0.04	0.14	-0.05	0.58	0.57	1
13	0.32	0.00	-0.20	-0.02	0.34	0.27		
14	0.30	-0.03	0.01	**0.55**	0.00	0.40	0.25	4
15	0.16	0.02	**0.58**	0.07	-0.10	0.37	0.42	3
16	0.18	0.06	-0.15	0.03	**0.46**	0.30	0.28	5
17	0.08	0.21	-0.02	0.06	0.35	0.23		
18	0.28	0.08	0.26	-0.13	0.22	0.27		
19	**0.55**	0.06	0.07	0.01	0.30	0.50	0.25	1
20	**0.71**	0.00	-0.04	0.04	-0.02	0.50	0.67	1
21	**0.54**	0.05	0.02	0.03	0.29	0.47	0.25	1
22	0.12	-0.14	0.33	-0.13	-0.24	0.19		
23	-0.10	**0.44**	0.01	0.39	0.01	0.39	0.05	
24	-0.09	0.26	0.22	**0.51**	0.03	0.44	0.25	4
25	-0.05	**0.47**	0.17	0.28	-0.03	0.36	0.19	
26	0.03	**0.48**	-0.03	0.11	0.08	0.29	0.37	2
27	-0.06	**0.47**	0.07	0.17	0.24	0.36	0.23	2
28	0.07	0.21	-0.02	-0.19	0.02	0.08		
29	0.00	**0.43**	0.09	0.22	-0.04	0.28	0.21	2
30	0.05	0.03	-0.09	**0.69**	-0.01	0.49	0.60	4
31	**0.66**	0.03	0.12	0.04	0.17	0.55	0.49	1
32	0.10	0.08	-0.25	-0.12	-0.19	0.14		
33	0.04	**0.60**	-0.02	0.13	0.01	0.43	0.47	2
34	0.13	0.37	-0.24	0.14	-0.28	0.33		
35	0.02	**0.70**	-0.19	-0.01	-0.23	0.56	0.47	2
36	0.18	0.01	**0.60**	-0.06	0.06	0.42	0.42	3
37	-0.03	**0.65**	-0.05	0.09	-0.06	0.43	0.56	2
38	-0.06	0.06	0.10	0.10	0.21	0.08		
39	0.14	0.06	-0.20	0.00	**0.47**	0.30	0.27	5
40	0.14	0.35	0.03	-0.04	0.32	0.32		

Note. The extraction method was principal component analyses with an oblique rotation. [1]Selection based on component loading rules (primary loading >.40; secundary loading <.30, difference primary - secundary loading >.20). Results are based on the pattern matrix. Results from the structural matrix did not diverge based on the final model obtained. Item number corresponds with the item number in Table 7. h^2 = communalities. Δ primary - secundary loading = difference between the primary and secundary loading.

Table 9 Continued

Results of the Principal Component Analyses of the Five Component Solution in the General Population Sample (N = 427)

Item Nr.	Component Loadings					h^2	Δ Primary-Secondary Loading	Selected Item based on Component Loading Rules[1]
	1	2	3	4	5			
41	0.32	0.18	-0.26	-0.28	-0.24	0.34		
42	0.07	0.23	-0.32	-0.13	-0.15	0.22		
43	0.17	0.09	**0.58**	0.05	0.01	0.40	0.41	3
44	-0.02	**0.66**	0.02	-0.09	0.25	0.53	0.41	2
45	0.06	0.03	0.02	0.03	0.36	0.16		
46	0.10	**0.68**	0.10	0.00	0.10	0.57	0.58	2
47	0.00	-0.05	-0.43	-0.06	0.12	0.19	0.31	3
48	0.14	-0.14	-0.42	0.34	-0.12	0.30	0.08	
49	**0.70**	0.06	0.18	-0.01	0.10	0.61	0.52	1
50	0.17	0.19	-0.45	-0.11	-0.08	0.31	0.26	3
51	0.11	0.38	-0.29	-0.18	0.04	0.29		
52	0.02	-0.04	-0.29	0.25	0.37	0.24		
53	-0.07	-0.02	0.25	0.00	0.32	0.18		
54	**0.80**	-0.08	0.03	0.05	-0.14	0.59	0.66	1
55	-0.04	**0.54**	-0.09	-0.04	-0.14	0.29	0.40	2
56	**0.49**	0.08	-0.08	-0.21	-0.05	0.30	0.28	1
57	0.09	0.04	0.01	**0.60**	0.07	0.40	0.51	4
58	**0.63**	0.06	-0.18	-0.08	-0.11	0.44	0.45	1
Coefficient Alpha	.89 (95% CI = .87-.91)	.83 (95% CI = .80-.85)	.69 (95% CI = .64-.74)	.66 (95% CI = .60-.71)	-			
Proportion of variance accounted for (%)	11	10	6	4	4			

Note. The extraction method was principal component analyses with an oblique rotation. [1]Selection based on component loading rules (primary loading >.40; secundary loading <.30, difference primary - secundary loading >.20). Results are based on the pattern matrix. Results from the structural matrix did not diverge based on the final model obtained. Item number corresponds with the item number in Table 7. h^2 = communalities. Δ primary - secundary loading = difference between the primary and secundary loading.

Table 10

Results of the Principal Component Analyses of the Six Component Solution in the General Population Sample (N = 427)

Item Nr.	Component Loadings						h^2	ΔPrimary-Secondary Loading	Selected Item based on Component Loading Rules[1]
	1	2	3	4	5	6			
1	0.07	**0.68**	0.12	-0.04	-0.02	-0.04	0.51	0.56	2
2	0.20	-0.01	-0.25	0.39	0.11	0.17	0.33		
3	**0.62**	0.09	0.09	0.05	-0.11	0.04	0.47	0.51	1
4	0.09	0.00	**0.60**	-0.04	0.00	-0.13	0.40	0.47	3
5	**0.70**	-0.05	-0.02	0.09	0.06	0.00	0.49	0.61	1
6	0.08	**0.67**	0.00	-0.04	-0.10	0.06	0.49	0.57	2
7	**0.78**	-0.04	0.07	0.00	0.04	0.07	0.64	0.71	1
8	0.07	-0.01	0.01	-0.19	-0.05	0.22	0.10		
9	0.11	0.29	0.31	0.10	-0.15	0.24	0.32		
10	0.23	0.28	-0.16	0.13	-0.03	0.24	0.31		
11	0.16	0.39	-0.14	-0.28	0.20	-0.24	0.39		
12	**0.73**	0.09	0.01	-0.02	0.11	-0.08	0.60	0.62	1
13	0.15	-0.04	0.04	0.24	0.14	**0.51**	0.40	0.27	6
14	0.21	-0.04	0.09	0.05	**0.60**	0.03	0.44	0.39	5
15	0.08	0.00	**0.62**	-0.02	0.12	-0.07	0.43	0.50	3
16	0.15	0.05	-0.12	-0.12	0.05	**0.45**	0.30	0.30	6
17	0.14	0.22	-0.12	-0.27	0.00	0.25	0.25		
18	0.16	0.05	0.38	0.06	-0.03	0.32	0.34		
19	**0.55**	0.05	0.04	-0.15	-0.01	0.26	0.51	0.29	1
20	**0.73**	0.00	-0.05	0.02	0.02	-0.03	0.52	0.68	1
21	**0.54**	0.05	0.00	-0.13	0.02	0.25	0.47	0.29	1
22	0.08	-0.15	0.36	0.09	-0.10	-0.20	0.20		
23	-0.07	**0.45**	-0.04	-0.13	0.34	-0.05	0.39	0.11	
24	-0.06	0.28	0.12	-0.26	**0.44**	-0.09	0.44	0.16	
25	0.03	**0.48**	0.03	-0.24	0.19	-0.15	0.39	0.24	2
26	-0.01	**0.47**	0.03	0.05	0.14	0.12	0.30	0.33	2
27	-0.05	**0.47**	0.03	-0.18	0.15	0.18	0.36	0.29	2
28	0.06	0.21	0.01	0.07	-0.17	0.06	0.08		
29	-0.01	**0.43**	0.10	-0.01	0.22	-0.04	0.28	0.21	2
30	-0.02	0.03	-0.02	0.01	**0.73**	0.01	0.54	0.70	5
31	**0.67**	0.03	0.08	-0.13	0.02	0.13	0.56	0.54	1
32	0.09	0.07	-0.18	0.25	-0.09	-0.10	0.14		
33	0.02	**0.59**	0.03	0.05	0.15	0.05	0.43	0.44	2
34	0.16	0.37	-0.20	0.19	0.13	-0.24	0.34		
35	0.04	**0.70**	-0.14	0.20	-0.02	-0.17	0.56	0.50	2
36	0.09	-0.01	**0.64**	-0.06	0.00	0.09	0.48	0.55	3
37	-0.05	**0.64**	0.01	0.10	0.11	-0.01	0.44	0.53	2
38	0.12	0.10	-0.19	-0.51	-0.09	-0.04	0.27	0.32	4
39	0.06	0.05	-0.09	0.00	0.08	**0.53**	0.33	0.44	6
40	0.03	0.32	0.16	0.06	0.05	**0.41**	0.37	0.09	

Note. The extraction method was principal component analyses with an oblique rotation.
[1]Selection based on component loading rules (primary loading >.40; secundary loading <.30, difference primary - secundary loading >.20). Results are based on the pattern matrix. Results from the structural matrix did not diverge based on the final model obtained. Item number corresponds with the item number in Table 7. h^2 = communalities. Δ primary - secundary loading = difference between the primary and secundary loading.

Table 10 Continued

Results of the Principal Component Analyses of the Six Component Solution in the General Population Sample (N = 427)

Item Nr.	Component Loadings						h^2	Δ Primary-Secondary Loading	Selected Item based on Component Loading Rules[1]
	1	2	3	4	5	6			
41	0.25	0.15	-0.07	**0.44**	-0.18	-0.05	0.35	0.19	
42	-0.01	0.21	-0.12	**0.41**	-0.02	0.03	0.25	0.20	4
43	0.05	0.06	**0.66**	0.01	0.14	0.07	0.50	0.52	3
44	-0.01	**0.66**	0.01	-0.08	-0.10	0.25	0.53	0.41	2
45	0.03	0.02	0.02	-0.14	0.04	0.35	0.16		
46	0.12	**0.68**	0.07	-0.08	-0.03	0.08	0.58	0.56	2
47	-0.11	-0.07	-0.23	0.31	0.06	0.28	0.25		
48	0.05	-0.16	-0.25	0.30	**0.43**	0.00	0.33	0.13	
49	**0.71**	0.05	0.14	-0.10	-0.03	0.06	0.62	0.57	1
50	0.07	0.17	-0.22	**0.45**	0.02	0.13	0.35	0.23	4
51	0.02	0.36	-0.10	0.33	-0.07	0.20	0.32		
52	0.14	-0.01	-0.47	-0.36	0.12	0.19	0.34	0.11	
53	0.02	-0.01	0.05	-0.43	-0.10	0.14	0.22	0.29	4
54	**0.83**	-0.08	0.00	0.03	0.02	-0.16	0.62	0.67	1
55	-0.05	**0.53**	-0.02	0.18	-0.02	-0.07	0.29	0.35	2
56	**0.49**	0.07	-0.04	0.14	-0.20	0.00	0.31	0.29	1
57	-0.02	0.03	0.11	0.03	**0.67**	0.11	0.48	0.56	5
58	**0.62**	0.05	-0.12	0.19	-0.06	-0.04	0.45	0.43	1
Coefficient Alpha	.89 (95% CI = .87-.91)	.83 (95% CI = .80-.85)	.69 (95% CI = .64-.74)	.41 (95% CI = .32-.50)	.66 (95% CI = .60-.71)	.44 (95% CI = .34-.52)			
Proportion of variance accounted for (%)	11	10	5	4	4	4			

Note. The extraction method was principal component analyses with an oblique rotation.
[1]Selection based on component loading rules (primary loading >.40; secondary loading <.30, difference primary - secundary loading >.20). Results are based on the pattern matrix. Results from the structural matrix did not diverge based on the final model obtained. Item number corresponds with the item number in Table 7. h^2 = communalities. Δ primary - secundary loading = difference between the primary and secundary loading.

Table 11

Pattern and Structure Matrix of the Four Component Solution of the Principal Component Analyses in the General Population Sample (N = 427)

Item nr.	Pattern Matrix Component				h^2	Structure Matrix Component			
	1	2	3	4		1	2	3	4
1	0.04	0.68	0.11	-0.01	0.49	0.28	0.69	-0.06	0.19
2	0.29	0.01	-0.47	-0.01	0.29	0.13	0.03	0.50	0.01
3	0.63	0.09	0.04	-0.12	0.46	0.66	0.31	-0.07	-0.03
4	0.10	0.00	0.54	-0.06	0.31	0.10	0.05	-0.52	0.07
5	0.70	-0.05	-0.10	0.04	0.47	0.67	0.19	0.02	0.11
6	0.07	0.68	0.02	-0.07	0.48	0.31	0.71	0.00	0.10
7	0.81	-0.04	0.04	0.03	0.64	0.79	0.23	-0.11	0.08
8	0.13	0.01	0.15	0.01	0.04	0.15	0.12	-0.23	-0.02
9	0.24	0.32	0.22	-0.23	0.28	0.34	0.39	-0.21	-0.12
10	0.31	0.30	-0.22	-0.06	0.29	0.34	0.43	0.12	0.02
11	0.01	0.36	0.01	0.33	0.28	0.14	0.39	-0.04	0.45
12	0.69	0.08	-0.01	0.12	0.55	0.72	0.33	-0.03	0.23
13	0.42	0.02	-0.11	0.02	0.19	0.36	0.16	0.04	0.06
14	0.30	-0.03	-0.01	0.54	0.38	0.31	0.17	-0.07	0.59
15	0.13	0.01	0.53	0.05	0.32	0.11	0.05	-0.52	0.15
16	0.30	0.10	-0.02	0.08	0.13	0.31	0.24	-0.08	0.10
17	0.18	0.24	0.08	0.10	0.15	0.23	0.32	-0.14	0.17
18	0.34	0.09	0.31	-0.12	0.25	0.38	0.22	-0.29	-0.06
19	0.63	0.08	0.14	0.03	0.47	0.67	0.32	-0.17	0.07
20	0.70	-0.01	-0.08	0.03	0.49	0.59	0.20	0.04	0.11
21	0.62	0.07	0.08	0.06	0.44	0.65	0.31	-0.14	0.10
22	0.05	-0.16	0.25	-0.16	0.12	0.04	-0.17	-0.12	-0.19
23	-0.10	0.44	0.00	0.38	0.39	0.07	0.47	-0.01	0.50
24	-0.08	0.27	0.22	0.50	0.43	0.09	0.34	-0.20	0.57
25	-0.06	0.47	0.14	0.27	0.35	0.15	0.49	-0.13	0.40
26	0.04	0.49	-0.02	0.11	0.29	0.18	0.52	-0.02	0.23
27	0.01	0.49	0.13	0.19	0.34	0.20	0.54	-0.15	0.30
28	0.07	0.21	-0.02	-0.19	0.08	0.05	0.18	0.00	-0.11
29	-0.02	0.43	0.07	0.21	0.26	0.16	0.46	-0.06	0.32
30	0.05	0.04	-0.10	0.69	0.49	0.09	0.17	0.00	0.70
31	0.70	0.04	0.14	0.05	0.55	0.74	0.31	-0.17	0.09
32	0.05	0.06	-0.31	-0.13	0.13	-0.02	-0.01	0.40	-0.15
33	0.04	0.60	-0.04	0.12	0.43	0.24	0.62	0.05	0.29
34	0.05	0.35	-0.34	0.11	0.27	0.12	0.27	0.51	0.18
35	-0.05	0.68	-0.27	-0.04	0.52	0.16	0.54	0.44	0.10
36	0.19	0.02	0.60	-0.07	0.41	0.24	0.13	-0.64	0.04
37	-0.05	0.64	-0.09	0.08	0.42	0.17	0.61	0.15	0.26
38	0.00	0.08	0.16	0.12	0.06	0.05	0.11	-0.20	0.16
39	0.27	0.10	-0.06	0.06	0.11	0.26	0.24	-0.03	0.08
40	0.22	0.37	0.11	-0.01	0.26	0.36	0.46	-0.10	0.08
41	0.25	0.15	-0.34	-0.30	0.30	0.11	0.11	0.43	-0.14
42	0.03	0.22	-0.37	-0.15	0.21	0.00	0.11	0.45	-0.07
43	0.17	0.09	0.56	0.04	0.38	0.25	0.17	-0.64	0.19
44	0.05	0.69	0.07	-0.07	0.49	0.29	0.72	-0.08	0.11
45	0.17	0.06	0.12	0.07	0.06	0.19	0.15	-0.17	0.06
46	0.13	0.69	0.10	0.00	0.57	0.37	0.75	-0.09	0.16
47	0.03	-0.05	-0.38	-0.04	0.15	-0.09	-0.09	0.38	-0.06
48	0.11	-0.16	-0.45	0.34	0.29	0.02	-0.08	0.49	0.27
49	0.72	0.06	0.17	-0.01	0.60	0.76	0.31	-0.19	0.05
50	0.15	0.18	-0.48	-0.11	0.31	0.02	0.13	0.51	-0.05
51	0.12	0.38	-0.29	-0.17	0.28	0.16	0.32	0.33	-0.09
52	0.12	-0.01	-0.18	0.29	0.12	0.07	0.09	0.06	0.26
53	0.02	0.00	0.34	0.03	0.12	0.11	0.06	-0.35	0.07
54	0.76	-0.10	-0.05	0.03	0.53	0.67	0.12	0.01	0.13
55	-0.08	0.53	-0.14	-0.06	0.27	0.10	0.47	0.21	0.04
56	0.47	0.07	-0.11	-0.22	0.29	0.41	0.18	0.05	-0.12
57	0.11	0.05	0.01	0.60	0.40	0.16	0.22	-0.11	0.62
58	0.60	0.04	-0.24	-0.09	0.42	0.61	0.20	0.17	0.02

Note. Item number corresponds with the item number in Table 7. h^2 = communalities.

Table 12

Pattern and Structure Matrix of the Five Component Solution of the Principal Component Analyses in the General Population Sample (N = 427)

Item nr.	Pattern Matrix Component					h^2	Structure Matrix Component				
	1	2	3	4	5		1	2	3	4	5
1	0.05	0.68	0.14	0.01	-0.02	0.503	0.29	0.70	-0.08	0.18	0.04
2	0.29	0.02	-0.46	-0.01	-0.01	0.295	0.12	0.03	0.52	0.02	0.09
3	0.63	0.10	0.07	-0.12	0.01	0.464	0.66	0.31	-0.09	-0.03	0.15
4	0.14	0.02	0.60	-0.03	-0.14	0.374	0.12	0.05	-0.55	0.06	-0.10
5	0.71	-0.04	-0.06	0.05	-0.03	0.486	0.69	0.20	0.01	0.11	0.06
6	0.05	0.67	0.02	-0.07	0.08	0.485	0.30	0.71	-0.01	0.10	0.11
7	0.79	-0.03	0.05	0.03	0.06	0.641	0.79	0.23	-0.11	0.08	0.22
8	0.06	-0.01	0.07	-0.02	0.28	0.098	0.11	0.09	-0.21	-0.01	0.27
9	0.20	0.32	0.19	-0.24	0.14	0.285	0.31	0.38	-0.21	-0.11	0.22
10	0.27	0.29	-0.25	-0.08	0.17	0.311	0.31	0.41	0.14	0.03	0.30
11	0.03	0.36	0.05	0.35	-0.07	0.303	0.18	0.42	-0.07	0.44	-0.19
12	0.71	0.09	0.04	0.14	-0.05	0.578	0.74	0.34	-0.05	0.23	0.10
13	0.32	0.00	-0.20	-0.02	0.34	0.268	0.29	0.12	0.08	0.09	0.48
14	0.30	-0.03	0.01	0.55	0.00	0.397	0.31	0.17	-0.08	0.59	0.07
15	0.16	0.02	0.58	0.07	-0.10	0.372	0.12	0.05	-0.53	0.14	-0.02
16	0.18	0.06	-0.15	0.03	0.46	0.295	0.23	0.20	-0.04	0.13	0.48
17	0.08	0.21	-0.02	0.06	0.35	0.228	0.19	0.30	-0.12	0.18	0.28
18	0.28	0.08	0.26	-0.13	0.22	0.267	0.34	0.19	-0.27	-0.05	0.34
19	0.55	0.06	0.07	0.01	0.30	0.5	0.63	0.29	-0.15	0.09	0.44
20	0.71	0.00	-0.04	0.04	-0.02	0.501	0.60	0.21	0.02	0.10	0.09
21	0.54	0.05	0.02	0.03	0.29	0.465	0.61	0.29	-0.12	0.12	0.39
22	0.12	-0.14	0.33	-0.13	-0.24	0.189	0.08	-0.15	-0.15	-0.20	-0.19
23	-0.10	0.44	0.01	0.39	0.01	0.388	0.07	0.47	-0.02	0.49	-0.02
24	-0.09	0.26	0.22	0.51	0.03	0.435	0.09	0.35	-0.21	0.56	-0.03
25	-0.05	0.47	0.17	0.28	-0.03	0.363	0.17	0.50	-0.16	0.39	-0.06
26	0.03	0.48	-0.03	0.11	0.08	0.29	0.16	0.52	-0.02	0.23	0.12
27	-0.06	0.47	0.07	0.17	0.24	0.359	0.17	0.52	-0.15	0.31	0.22
28	0.07	0.21	-0.02	-0.19	0.02	0.082	0.05	0.18	0.00	-0.11	0.01
29	0.00	0.43	0.09	0.22	-0.04	0.275	0.17	0.47	-0.09	0.31	-0.04
30	0.05	0.03	-0.09	0.69	-0.01	0.492	0.08	0.17	0.00	0.71	0.04
31	0.66	0.03	0.12	0.04	0.17	0.551	0.71	0.30	-0.17	0.10	0.32
32	0.10	0.08	-0.25	-0.12	-0.19	0.14	0.01	0.01	0.39	-0.16	-0.12
33	0.04	0.60	-0.02	0.13	0.01	0.431	0.23	0.62	0.05	0.30	0.13
34	0.13	0.37	-0.24	0.14	-0.28	0.334	0.18	0.31	0.48	0.16	-0.26
35	0.02	0.70	-0.19	-0.01	-0.23	0.559	0.19	0.57	0.41	0.08	-0.15
36	0.18	0.01	0.60	-0.06	0.06	0.419	0.22	0.11	-0.64	0.05	0.17
37	-0.03	0.65	-0.05	0.09	-0.06	0.432	0.18	0.63	0.13	0.25	-0.03
38	-0.06	0.06	0.10	0.10	0.21	0.083	0.04	0.10	-0.20	0.16	0.04
39	0.14	0.06	-0.20	0.00	0.47	0.295	0.18	0.20	0.01	0.12	0.48
40	0.14	0.35	0.03	-0.04	0.32	0.316	0.31	0.43	-0.08	0.10	0.37
41	0.32	0.18	-0.26	-0.28	-0.24	0.337	0.15	0.14	0.42	-0.15	-0.16
42	0.07	0.23	-0.32	-0.13	-0.15	0.217	0.02	0.13	0.44	-0.08	-0.13
43	0.17	0.09	0.58	0.05	0.01	0.397	0.23	0.16	-0.64	0.19	0.16
44	-0.02	0.66	0.02	-0.09	0.25	0.527	0.25	0.70	-0.07	0.12	0.28
45	0.06	0.03	0.02	0.03	0.36	0.155	0.14	0.12	-0.14	0.08	0.34
46	0.10	0.68	0.10	0.00	0.10	0.571	0.36	0.75	-0.11	0.16	0.14
47	0.00	-0.05	-0.43	-0.06	0.12	0.193	-0.12	-0.10	0.41	-0.05	0.11
48	0.14	-0.14	-0.42	0.34	-0.12	0.295	0.03	-0.07	0.49	0.27	-0.09
49	0.70	0.06	0.18	-0.01	0.10	0.605	0.75	0.31	-0.19	0.06	0.25
50	0.17	0.19	-0.45	-0.11	-0.08	0.309	0.02	0.13	0.52	-0.05	-0.01
51	0.11	0.38	-0.29	-0.18	0.04	0.288	0.13	0.31	0.35	-0.08	0.20
52	0.02	-0.04	-0.29	0.25	0.37	0.243	0.02	0.06	0.09	0.28	0.28
53	-0.07	-0.02	0.25	0.00	0.32	0.18	0.07	0.03	-0.33	0.08	0.25
54	0.80	-0.08	0.03	0.05	-0.14	0.588	0.71	0.15	-0.02	0.11	-0.07
55	-0.04	0.54	-0.09	-0.04	-0.14	0.288	0.12	0.48	0.19	0.03	-0.06
56	0.49	0.08	-0.08	-0.21	-0.05	0.3	0.41	0.18	0.05	-0.12	0.12
57	0.09	0.04	0.01	0.60	0.07	0.398	0.13	0.21	-0.11	0.63	0.14
58	0.63	0.06	-0.18	-0.08	-0.11	0.443	0.64	0.22	0.15	0.01	-0.01

Note. Item number corresponds with the item number in Table 7. h^2 = communalities.

Table 13

Pattern and Structure Matrix of the Six Component Solution of the Principal Component Analyses in the General Population Sample (N = 427)

Item nr.	Pattern Matrix Component						h^2	Structure matrix Component					
	1	2	3	4	5	6		1	2	3	4	5	6
1	0.07	0.68	0.12	-0.04	-0.02	-0.04	0.505	0.29	0.69	0.14	-0.07	0.14	0.10
2	0.20	-0.01	-0.25	0.39	0.11	0.17	0.329	0.23	0.10	-0.29	0.44	0.08	0.19
3	0.62	0.09	0.09	0.05	-0.11	0.04	0.467	0.66	0.28	0.16	0.07	-0.02	0.22
4	0.09	0.00	0.60	-0.04	0.00	-0.13	0.396	0.13	0.02	0.61	-0.17	0.03	-0.06
5	0.70	-0.05	-0.02	0.09	0.06	0.00	0.489	0.69	0.19	0.05	0.12	0.12	0.17
6	0.08	0.67	0.00	-0.04	-0.10	0.06	0.492	0.30	0.69	0.02	-0.05	0.06	0.19
7	0.78	-0.04	0.07	0.00	0.04	0.07	0.644	0.80	0.23	0.17	0.02	0.11	0.28
8	0.07	-0.01	0.01	-0.19	-0.05	0.22	0.1	0.12	0.04	0.08	-0.19	-0.02	0.24
9	0.11	0.29	0.31	0.10	-0.15	0.24	0.32	0.30	0.33	0.31	0.05	-0.07	0.33
10	0.23	0.28	-0.16	0.13	-0.03	0.24	0.312	0.37	0.38	-0.14	0.16	0.04	0.33
11	0.16	0.39	-0.14	-0.28	0.20	-0.24	0.393	0.21	0.45	-0.07	-0.27	0.32	-0.14
12	0.73	0.09	0.01	-0.02	0.11	-0.08	0.596	0.75	0.35	0.10	-0.01	0.21	0.14
13	0.15	-0.04	0.04	0.24	0.14	0.51	0.401	0.31	0.12	0.05	0.22	0.14	0.55
14	0.21	-0.04	0.09	0.05	0.60	0.03	0.443	0.29	0.17	0.13	-0.03	0.62	0.11
15	0.08	0.00	0.62	-0.02	0.12	-0.07	0.425	0.15	0.05	0.63	-0.16	0.15	0.01
16	0.15	0.05	-0.12	-0.12	0.05	0.45	0.296	0.27	0.19	-0.04	-0.10	0.10	0.50
17	0.14	0.22	-0.12	-0.27	0.00	0.25	0.249	0.25	0.31	-0.02	-0.25	0.09	0.32
18	0.16	0.05	0.38	0.06	-0.03	0.32	0.336	0.31	0.15	0.41	-0.02	0.01	0.39
19	0.55	0.05	0.04	-0.15	-0.01	0.26	0.505	0.64	0.28	0.17	-0.15	0.08	0.42
20	0.73	0.00	-0.05	0.02	0.02	-0.03	0.519	0.72	0.24	0.04	0.06	0.09	0.16
21	0.54	0.05	0.00	-0.13	0.02	0.25	0.471	0.62	0.27	0.11	-0.12	0.11	0.41
22	0.08	-0.15	0.36	0.09	-0.10	-0.20	0.198	0.02	-0.18	0.33	0.04	-0.12	-0.17
23	-0.07	0.45	-0.04	-0.13	0.34	-0.05	0.388	0.09	0.49	-0.01	-0.17	0.44	0.00
24	-0.06	0.28	0.12	-0.26	0.44	-0.09	0.435	0.06	0.35	0.18	-0.34	0.52	-0.04
25	0.03	0.48	0.03	-0.24	0.19	-0.15	0.39	0.17	0.52	0.09	-0.27	0.32	-0.06
26	-0.01	0.47	0.03	0.05	0.14	0.12	0.298	0.20	0.52	0.04	0.01	0.24	0.19
27	-0.05	0.47	0.03	-0.18	0.15	0.18	0.359	0.17	0.52	0.08	-0.22	0.27	0.25
28	0.06	0.21	0.01	0.07	-0.17	0.06	0.082	0.13	0.20	0.00	0.08	-0.13	0.10
29	-0.01	0.43	0.10	-0.01	0.22	-0.04	0.277	0.15	0.47	0.11	-0.07	0.31	0.03
30	-0.02	0.03	-0.02	0.01	0.73	0.01	0.537	0.07	0.19	0.00	-0.06	0.73	0.02
31	0.67	0.03	0.08	-0.13	0.02	0.13	0.561	0.72	0.28	0.20	-0.12	0.11	0.32
32	0.09	0.07	-0.18	0.25	-0.09	-0.10	0.14	0.07	0.06	-0.23	0.30	-0.09	-0.09
33	0.02	0.59	0.03	0.05	0.15	0.05	0.434	0.25	0.64	0.03	0.02	0.27	0.15
34	0.16	0.37	-0.20	0.19	0.13	-0.24	0.34	0.21	0.41	-0.24	0.22	0.19	-0.16
35	0.04	0.70	-0.14	0.20	-0.02	-0.17	0.563	0.21	0.68	-0.19	0.22	0.11	-0.07
36	0.09	-0.01	0.64	-0.06	0.00	0.09	0.476	0.19	0.04	0.67	-0.20	0.04	0.17
37	-0.05	0.64	0.01	0.10	0.11	-0.01	0.435	0.17	0.64	-0.02	0.07	0.23	0.07
38	0.12	0.10	-0.19	-0.51	-0.09	-0.04	0.267	0.09	0.12	-0.07	-0.46	-0.01	0.00
39	0.06	0.05	-0.09	0.00	0.08	0.53	0.325	0.21	0.16	-0.04	0.00	0.10	0.55
40	0.03	0.32	0.16	0.06	0.05	0.41	0.372	0.27	0.41	0.19	0.00	0.13	0.48
41	0.25	0.15	-0.07	0.44	-0.18	-0.05	0.349	0.27	0.18	-0.14	0.48	-0.17	0.02
42	-0.01	0.21	-0.12	0.41	-0.02	0.03	0.25	0.06	0.20	-0.21	0.44	-0.02	0.04
43	0.05	0.06	0.66	0.01	0.14	0.07	0.498	0.19	0.12	0.68	-0.15	0.18	0.15
44	-0.01	0.66	0.01	-0.08	-0.10	0.25	0.528	0.26	0.67	0.05	-0.10	0.06	0.34
45	0.03	0.02	0.02	-0.14	0.04	0.35	0.159	0.14	0.10	0.08	-0.15	0.08	0.36
46	0.12	0.68	0.07	-0.08	-0.03	0.08	0.575	0.37	0.73	0.12	-0.11	0.15	0.22
47	-0.11	-0.07	-0.23	0.31	0.06	0.28	0.246	-0.07	-0.06	-0.29	0.34	0.00	0.22
48	0.05	-0.16	-0.25	0.30	0.43	0.00	0.334	0.03	-0.05	-0.29	0.32	0.36	-0.02
49	0.71	0.05	0.14	-0.10	-0.03	0.06	0.617	0.76	0.29	0.26	-0.10	0.07	0.27
50	0.07	0.17	-0.22	0.45	0.02	0.13	0.347	0.15	0.20	-0.30	0.49	0.01	0.14
51	0.02	0.36	-0.10	0.33	-0.07	0.20	0.319	0.19	0.37	-0.15	0.35	-0.02	0.25
52	0.14	-0.01	-0.47	-0.36	0.12	0.19	0.339	0.13	0.10	-0.35	-0.27	0.16	0.20
53	0.02	-0.01	0.05	-0.43	-0.10	0.14	0.223	0.04	0.01	0.15	-0.43	-0.05	0.15
54	0.83	-0.08	0.00	0.03	0.02	-0.16	0.615	0.76	0.17	0.09	0.07	0.09	0.05
55	-0.05	0.53	-0.02	0.18	-0.02	-0.07	0.289	0.11	0.50	-0.07	0.17	0.08	-0.01
56	0.49	0.07	-0.04	0.14	-0.20	0.00	0.306	0.49	0.19	-0.01	0.18	-0.14	0.13
57	-0.02	0.03	0.11	0.03	0.67	0.11	0.484	0.11	0.19	0.13	-0.06	0.68	0.14
58	0.62	0.05	-0.12	0.19	-0.06	-0.04	0.448	0.61	0.23	-0.09	0.25	0.00	0.12

Note. Item number corresponds with the item number in Table 7. h^2 = communalities.

4. Mean Differences Men Who Sexually Offended Against Children

online supplementary material 4 is complementary to the result section in the full paper. It contains the mean differences between the several population samples with the sample of men who sexually offended only including men who sexually offended against children.

Table 14

Mean Differences Between the Different Population Samples of the Factors of the Implicit Theories of Sexual Offense Questionnaire

	M (*SD*)				
		(sub)Clinical Sample			
	General Population Sample (*n* = 427)	Men Who Violently Offended (*n* = 21)	Pedophilia-supportive Forum Users (*n* = 20)	Men Who Sexually Offend Against Children (*n* = 19)	Difference
F1 Child	2.02 (0.77)	1.99 (0.91)	2.60 (1.13)	2.12 (1.18)	$H(3) = 5.669$, $p = .129$, $\varepsilon^2 = .01$
F2 Women	2.25 (0.70)	2.65 (0.80)	2.24 (0.58)	2.26 (0.72)	$H(3) = 5.855$, $p = .119$, $\varepsilon^2 = .01$
F3 SSDI	4.62 (0.62)	4.36 (0.52)	4.61 (0.50)	4.42 (0.68)	**$H(3)=11.286$, $p = .010$, $\varepsilon^2 = .02$**
F4 Uncontr.	2.97 (0.82)	3.42 (0.68)	3.09 (0.73)	3.37 (0.98)	**$H(3)= 9.055$, $p = .029$, $\varepsilon^2 = .03$**
IM	0.35 (0.23)	0.34 (0.19)	0.38 (0.28)	0.37 (0.18)	$H(3)= 0.781$, $p = .085$, $\varepsilon^2 = .00$
SDE	0.28 (0.22)	0.34 (0.27)	0.20 (0.14)	0.31 (0.16)	$H(3)= 4.468$, $p = .215$, $\varepsilon^2 = .00$

Note. While Kruskal Wallis analyses indicated significant group differences on F4 Uncontrollability, when comparing the specific population samples, no significant differences were found after applying Holm's correction (Holm, 1979) for multiple comparisons. SSDI = Social Sexual Desirability Index. Uncontr. = Uncontrollability. IM = Impression Management. SDE = Self-Deceptive Enhancement. Significant values ($p \leq .05$) are in **bold**.

5. Sexual Interest and the ITSOQ, controlled for Social Desirability

Online supplementary material 4 is complementary to the result section in the full paper. It contains the correlational analyses between sexual interest and the factors of ITSOQ for the general and (sub)clinical population samples controlled for social desirability.

Table 15

Correlational Analyses Between Sexual Interest and the Factors of the Implicit Theory of Sexual Offense Questionnaire for the General Population Samples Controlled for Social Desirability

				Controlled for SSDI			Controlled for IM			Controlled for SDE		
Sexual Interest Towards…	*M* (*SD*)	Range	% Sexual Interest	F1 Child	F2 Women	F4 Uncontr.	F1 Child	F2 Women	F4 Uncontr.	F1 Child	F2 Women	F4 Uncontr.
...adult women	84.70 (25.40)	0 - 100	96.47	.05	.06	-.04	.06	.04	-.03	**.12**	.08	.00
...adult men	6.69 (19.60)	0 - 100	23.00	.07	**-.15**	.01	.07	**-.18**	-.01	.05	**-.15**	-.01
...girls age 14-16	11.00 (19.60)	0 - 100	41.65	**.31**	.08	-.02	**.30**	.03	-.03	**.30**	.08	-.02
...boys age 14-16	0.82 (4.86)	0 - 70	7.53	.05	-.07	-.04	.07	-.06	-.03	.03	-.08	-.04
...girls age 12-13	1.00 (3.72)	0 - 25	11.05									
...boys age 12-13	0.32 (3.05)	0 - 58	4.93									
...girls age ≤ 11	0.13 (0.85)	0 - 8	4.71									
...boys age ≤ 11	0.39 (4.31)	0 - 81	4.70									

Note. Spearman Rho analyses. For the general population sample it was not possible to look at the correlation between sexual interest with boys age 14-16, sexual interest towards girls/boys age 12-13, sexual interest towards girls/boys age ≤ 11 and the factors of the ITSO due to a general low score on these items. % sexual interest indicates any indication (% > 0) on the item. SSDI = Social Sexual Desirability Index. IM = Impressionamangement scale of the BIDR. SDE = Self-decelptive Enhancement scale of the BIDR. Uncontr. = Uncontrollabiliy. The *n* for the general population = 424/425. Significant values ($p \leq .05$) are in **bold**.

Table 16

Correlational Analyses Between Sexual Interest and the Factors of the Implicit Theory of Sexual Offense Questionnaire for the (sub)Clinical Population Samples Controlled for Social Desirability

				Controlled for SSDI			Controlled for IM			Controlled for SDE		
Sexual Interest Towards…	*M* (*SD*)	Range	% Sexual Interest	F1 Child	F2 Women	F4 Uncontr.	F1 Child	F2 Women	F4 Uncontr.	F1 Child	F2 Women	F4 Uncontr.
...adult women	58.10 (41.60)	0 - 100	87.76	-.12	**.35**	.08	-.10	**.35**	.05	-.02	**.36**	.20
...adult men	16.60 (29.20)	0 - 97	38.78	.28	-.04	-.03	**.32**	-.05	-.04	.24	-.02	-.11
...girls age 14-16	19.40 (32.20)	0 - 100	48.98	**.46**	.05	-.24	**.45**	.04	-.20	.48	.03	-.22
...boys age 14-16	19.60 (33.90)	0 - 100	38.78	**.42**	-.19	-.14	**.45**	-.19	-.14	**.39**	-.18	-.21
...girls age 12-13	16.60 (29.00)	0 - 100	39.58	**.36**	-.03	-.21	**.36**	-.03	-.19	.36	-.03	-.22
...boys age 12-13	21.30 (37.10)	0 - 100	36.73	**.36**	-.23	-.12	**.38**	-.23	-.12	**.32**	-.21	-.21
...girls age ≤ 11	19.20 (35.30)	0 - 100	32.65	**.33**	-.10	-.23	**.33**	-.11	-.22	**.32**	-.10	-.27
...boys age ≤ 11	22.10 (37.10)	0 - 100	34.69	**.36**	-.24	-.10	**.40**	-.25	-.11	**.34**	-.23	-.17

Note. Spearman Rho analyses. SSDI = Social Sexual Desirability Index. IM = Impressionamangement scale of the BIDR. SDE = Self-decelptive Enhancement scale of the BIDR. Uncontr. = Uncontrollabiliy. The n for the (sub)clinical population is lower due to the fact that the men who sexually offended from one inpatient clinic did not answer the questions concerning interest. The *n* for the (sub)clinical population = 48. Significant values ($p \leq .05$) are in **bold**.
